# A nanogram-sensitive workflow for oligonucleotide mass spectrometry using ion-pair-free nanoflow HILIC and RNase benchmarking

**DOI:** 10.1093/nar/gkag691

**Published:** 2026-07-14

**Authors:** Yuyang Qi, Chengkang Li, Nur Yesiltac-Tosun, Jannick Schicktanz, Leona Rusling, Steffen Kaiser, Samuel Wein, Stefanie Kaiser

**Affiliations:** Department of Pharmaceutical Chemistry, Goethe University Frankfurt, Frankfurt (Main) 60438, Germany; Department of Pharmaceutical Chemistry, Goethe University Frankfurt, Frankfurt (Main) 60438, Germany; Pediatric Cancer Metabolism Laboratory, Children’s Research Center, University of Zurich, Zurich 8008, Switzerland; Division of Oncology, University Children’s Hospital Zurich and Children’s Research Center, University of Zurich, Zurich 8008, Switzerland; Department of Pharmaceutical Chemistry, Goethe University Frankfurt, Frankfurt (Main) 60438, Germany; Department of Pharmaceutical Chemistry, Goethe University Frankfurt, Frankfurt (Main) 60438, Germany; Department of Pharmaceutical Chemistry, Goethe University Frankfurt, Frankfurt (Main) 60438, Germany; Mass Spectrometry Service Unit, Goethe-University Frankfurt, Max-von-Laue-Str. 9, Frankfurt (Main) 60438, Germany; Mass Spectrometry Service Unit, Goethe-University Frankfurt, Max-von-Laue-Str. 9, Frankfurt (Main) 60438, Germany; Applied Bioinformatics, Department of Computer Science, University of Tübingen, Tübingen 72074, Germany; OpenMS Inc., Erie, Pennsylvania 16502, United States; Department of Pharmaceutical Chemistry, Goethe University Frankfurt, Frankfurt (Main) 60438, Germany

## Abstract

RNA modifications regulate diverse cellular processes, yet comprehensive characterization of modified RNA sequences remains technically challenging. Mass spectrometry provides direct chemical information on RNA, but current oligonucleotide-based workflows typically require micrograms of RNA input and often rely on ion-pairing reagents for chromatographic separation, limiting their applicability to scarce or native RNA samples. Here, we establish a sensitive oligonucleotide mass spectrometry workflow that combines ion-pair-free nanoflow hydrophilic interaction liquid chromatography with systematic benchmarking of controlled RNA cleavage strategies. We compared RNase T1, RNase 4, and colicin E5 and evaluated how reaction conditions influence cleavage specificity, fragment length distribution, and terminal chemistries of RNA hydrolysates. The resulting workflow enables robust LC-MS/MS analysis using standard MS-compatible buffers and supports confident oligonucleotide identification through NucleicAcidSearchEngine (NASE) database searching. Using this approach, we achieved high sequence coverage from nanogram-scale RNA inputs, enabling modification analysis of 25–50 ng native yeast tRNA^Phe^ and sequence verification of 250 ng synthetic mRNA. Together, this work establishes a sensitive and broadly applicable platform for oligonucleotide mass spectrometry and provides practical guidance for RNase selection and digestion strategies. The method expands the applicability of RNA MS to low-input samples and supports future studies of RNA sequence and modification landscapes.

## Graphical Abstract

**Graphical Abstract:**
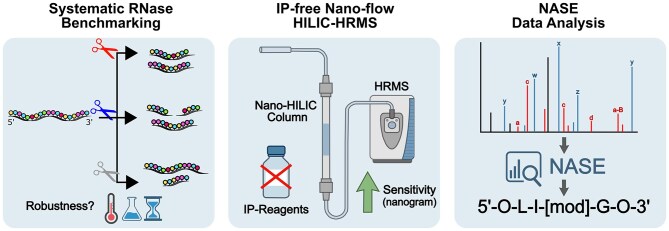

## Introduction

RNA modifications expand the functional diversity of the transcriptome and play central roles in cellular regulation. To date, ~150 different RNA modifications are known [1], which occur in all life forms and RNA species studied to date. RNA modifications are increasingly recognized to impact human health [2], disease prevention [3], and agricultural success [4]. The characterization of RNA modifications requires analytical approaches that provide both sequence context and direct chemical information. However, existing technologies do not yet robustly deliver this combined information for native RNA at low input levels. Consequently, comprehensive and site-specific analysis of modified RNAs remains a central challenge in RNA biology.

Two orthogonal concepts for RNA modification analysis have been established, namely mass spectrometry (MS) and sequencing. Both provide complementary information but also exhibit intrinsic limitations. Next-generation sequencing allows sensitive detection of nanogram amounts of RNA but relies on reverse transcription (RT) of RNA into complementary DNA (cDNA). RNA modifications are lost during cDNA synthesis, and only a few remain detectable through e.g. RT blocks or misincorporations [5, 6]. New reverse transcriptases address this particular challenge for tRNAs that have dense and chemically diverse modifications [7]. A similar sensitivity can be achieved by polymerase-chain reaction, which was recently used to detect tRNA-derived fragments from 100 ng of total RNA [8]. Third-generation sequencing, such as Oxford Nanopore sequencing, analyze RNA directly by measuring changes in electrical current as nucleotides pass through a pore. Although these approaches are rapidly evolving and show potential for direct modification detection (as often shown for pseudouridine [9]), their accuracy and interpretation remain dependent on signal modeling and training data [10, 11]. As a result, sequencing-based methods currently provide limited direct chemical resolution of RNA modifications.

MS is a valuable tool to analyze RNA modifications as it allows chemical characterization of RNA modifications [12]. Commonly, RNA is purified and hydrolyzed to the nucleoside building block with subsequent targeted MS analysis. While this approach provides valuable information about the absolute abundance and stoichiometry of RNA modifications [13–15], its key bottleneck is the loss of all sequence information. This fundamental limitation restricts its utility for comprehensive mapping of RNA modification landscapes.

To overcome this limitation, bottom-up oligonucleotide MS approaches have been developed. In these workflows, RNA is enzymatically cleaved into defined fragments using sequence-specific ribonucleases, followed by chromatographic separation and MS/MS analysis. This strategy preserves both sequence information and chemical modification content within individual fragments. Conceptually, bottom-up RNA MS parallels shotgun proteomics. The workflow relies on four key steps: controlled RNA fragmentation, chromatographic separation, mass spectrometric detection, and computational data analysis. First implementations of this approach date back to the introduction of electrospray ionization in the 1990s [16], and recent advances have enabled its application to increasingly complex RNA substrates [17–19] including modification mapping in transfer RNA (tRNA) and ribosomal RNA (rRNA) [20–25].

The analytical performance of oligonucleotide MS critically depends on the properties of the employed ribonucleases. Sequence coverage, fragment length distribution, and redundancy of overlapping fragments are directly determined by cleavage specificity. In contrast to proteomics, where broadly applicable proteases such as trypsin are available, no universally applicable RNase exists.

RNase T1 represents the most widely used enzyme and serves as a benchmark due to its well-defined cleavage 3′ of guanosine residues [17, 18, 26, 27]. This high specificity enables predictable fragmentation patterns but also results in relatively short fragments and incomplete sequence coverage. In addition, RNase T1 preferentially cleaves single-stranded regions, which can lead to missed cleavages in structured RNAs and thereby generate longer fragments [28]. While such missed cleavages can increase sequence information, they simultaneously expand the search space due to the growing number of possible fragment combinations, a challenge also encountered in proteomics [29].

Importantly, RNase T1 cleavage is influenced by RNA chemistry and modifications. For example, RNase T1 cleaves after inosine [30] but is inhibited by certain methylated guanosine derivatives [31]. This dependence on sequence context, structure, and chemical modifications is not unique to RNase T1 but represents a general challenge in enzymatic RNA fragmentation.

Consequently, the choice of RNase directly determines the accessibility of sequence regions and the interpretability of MS data. A conceptual classification of RNases is provided in Table 1, with a detailed overview in Supplementary Table S1. Importantly, no single RNase provides sufficient sequence coverage due to inherent trade-offs between cleavage frequency, fragment length, and sequence uniqueness. Different RNases generate complementary fragment sets, where frequently cleaving enzymes provide dense but redundant coverage, while more selective enzymes yield longer, more unique fragments. Current workflows often rely on a limited set of enzymes [17, 18, 27, 32, 33], and their substrate specificity is rarely characterized under varying experimental conditions.

**Table 1.** Conceptual classification of RNases used in this study

Class	Example RNases	Key property
Base-specific	T1, A	Predictable but short fragments
Context-dependent	RNase 4	Variable cleavage
Motif-specific	Colicin E5, MazF	High specificity, limited coverage

In this context, RNase 4 and colicin E5 represent particularly interesting candidates. These enzymes may be useful to probe complementary aspects of sequence coverage and modification sensitivity. RNase 4 has been reported to preferentially cleave after uridine in a context-dependent manner [27, 32], yet its performance with respect to RNA structure, modifications, and robustness remains insufficiently characterized. Colicin E5, originally described as a tRNA anticodon nuclease targeting YG|UN motifs [34], is capable of cleaving even in the presence of the hypermodified nucleotide queuosine [35]. However, its behavior in less-structured or unmodified RNA contexts and its suitability for general oligonucleotide MS applications has not been assessed in detail [17].

Chromatographic separation represents a second major bottleneck in oligonucleotide MS. Reversed-phase liquid chromatography typically requires ion-pairing (IP) reagents to retain highly polar oligonucleotides [36]. However, these additives reduce LC-MS compatibility, suppress ionization efficiency, and complicate method transferability [37, 38]. To assess the current state of sensitivity in published IP–RP methods, reported injection amounts across different flow rates were summarized in Supplementary Table S2 and Supplementary Fig. S25. While scaling down to micro- and nanoflow ranges (<100 µl/min and <1 µl/min, respectively) improves sensitivity down to the low femtomole level for synthetic analytes, the analysis of complex RNA hydrolysates still frequently requires injecting micrograms of RNA. For nanoflow workflows, RNA injections of nanogram amounts have been reported [39].

MS facilities are concerned about ion suppression caused by IP reagents and alternative IP-free chromatography systems have been explored. One option is the use of RP chromatography without IP reagents. Although chromatographic separation is possible, the ionization of oligonucleotides >10 nt (nucleotides) is inefficient due to insufficient desolvation in the electrospray ion source [40]. Hydrophilic interaction liquid chromatography (HILIC) offers an alternative separation mechanism that is intrinsically compatible with MS-friendly solvents and was successfully used for oligonucleotide analysis [22, 41, 42]. Regarding separation, a recent report systematically benchmarked 10 different HILIC materials for analysis of oligonucleotides and found that separation is good for small RNA fragments smaller than 15 nt but inefficient for longer fragments [42]. Regarding sensitivity, the LOD was reported to be 13 ng using synthetic oligonucleotides (equals 2 pmol for a 20-nt long oligonucleotide) [41, 43]. Recently, a report showed that mobile phase additives aid oligonucleotide separation and ionization using the BEH amide chemistry [41]. To the best of our knowledge HILIC was, unlike IP–RP methods, not yet coupled with nanoflow, particularly in combination with controlled enzymatic fragmentation and high-sensitivity detection of native RNA hydrolysates.

Taken together, current approaches do not yet provide an integrated solution that combines high sensitivity, ion-pair-free chromatographic separation, and controlled, reproducible RNA fragmentation. This limitation restricts the application of oligonucleotide MS to low-abundance or biologically relevant RNA samples.

Here, we address these limitations by developing an ion-pair-free nanoflow HILIC-MS workflow for sequence-resolved analysis of RNA hydrolysates. We systematically benchmark the substrate specificity of multiple RNases, including RNase T1, RNase 4, and colicin E5, under varying experimental conditions to define their complementary cleavage behavior.

In addition, we establish a nanoflow HILIC separation compatible with MS-friendly solvents and integrate these advances into a unified analytical workflow. Using this approach, we achieve sequence-resolved analysis of RNA at nanogram input levels and demonstrate its applicability to native and synthetic RNA substrates.

While the workflow is compatible with modification-containing RNAs, we focus here on establishing sensitivity, robustness, and fragmentation control as key parameters for bottom–up RNA MS.

## Materials and methods

### Chemicals and reagents

All chemicals and reagents were purchased from Sigma–Aldrich (St. Louis, MO, USA) unless otherwise specified.

RNA and DNA oligonucleotides were purchased from Sigma–Aldrich or Dharmacon, as specified in Supplementary Table S2.

Firefly luciferase (FLuc) mRNA was received from NIST (The National Institute of Standards and Technology, RGTM 10202).

Liquid chromatography (tandem) mass spectrometry (LC-MS) grade acetonitrile (ACN; Art. No. HN40), Urea (3941.1), and Tris (4855.2) were obtained from Carl Roth (Karlsruhe, Germany). LC-MS grade ammonium acetate (84885.180P, HiPerSolv CHROMANORM®) and acetic acid (84874.180, HiPerSolv CHROMANORM®) were purchased from VWR (Darmstadt, Germany).

### Expression and purification of colicin E5

The methods used for overexpression and purification of colicin E5 were as described previously [44] and is detailed in the Supplementary data. Briefly, the expression plasmid pD454-CE5-Im5 (GenScript Biotech, Netherlands) was transformed into BL21(DE3) chemically competent cells. Transformed cells were plated on LB-agar containing 50 µg/ml kanamycin and incubated overnight at 37°C. A single colony was inoculated into TB medium supplemented with chloramphenicol (0.034 mg/ml) and kanamycin (0.1 mg/ml) and incubated overnight at 37°C. For large-scale expression, the culture was expanded, and protein overexpression was induced with 0.5 mM IPTG (Isopropyl β-D-1-thiogalactopyranoside) at OD_600_ = 1.5, followed by overnight incubation at 18°C. Cells were harvested by centrifugation (6000 rpm, 4°C, 15 min) and resuspended in lysis buffer {25 mM HEPES, pH 7.5, 500 mM NaCl, 25 mM imidazole, 5% glycerol, 0.5 mM TCEP [tris(2-carboxyethyl)phosphine]}. After sonication, nucleic acids were removed by polyethyleneimine precipitation, and the lysate was cleared by centrifugation (23 000 × *g*, 40 min). The supernatant was filtered (0.45 µm) and loaded onto a 5-ml IMAC column (ÄKTAprime system) pre-equilibrated with lysis buffer. The column was washed with 10 mM imidazole buffer, followed by sequential washes with refold buffer 1 (25 mM HEPES, pH 7.5, 25 mM NaCl, 5% glycerol, 0.5 mM TCEP) and refold buffer 2 (25 mM HEPES, pH 7.5, 500 mM NaCl, 5% glycerol, 0.5 mM TCEP). CE5 was eluted using a 0–300 mM imidazole gradient in IMAC elution buffer (25 mM HEPES, pH 7.5, 500 mM NaCl, 300 mM imidazole, 5% glycerol, 0.5 mM TCEP) (Supplementary Fig. S1). For further purification, CE5-containing fractions were pooled, concentrated, and subjected to size-exclusion chromatography (SEC) using a Superdex™ 75 column in SEC buffer (20 mM HEPES, pH 7.5, 250 mM NaCl, 0.5 mM TCEP). The purified colicin E5 protein was concentrated to 3.5 mg/ml, yielding ∼20 mg, and stored at –80°C.

### *In vitro* transcription of *Escherichia coli* tRNAIle

Plasmid *pPK1204* with the correctly sequenced tRNA^Ile^ insert was transformed into *E. coli* DH5α competent cells and cultured on a large scale to produce sufficient plasmid DNA. The plasmid DNA was isolated using a Qiafilter Plasmid Maxi Kit (Qiagen, Venlo, Netherlands) and linearized with HindIII according to manufacturer protocol. *In vitro* transcription (IVT) of tRNA^Ile^ was conducted using optimized concentrations of DNA template and magnesium acetate. For large-scale transcription, the process was performed under these optimized conditions for a duration of 4 h. Specifically, the conditions included 40 mM magnesium acetate and 100 ng/µl of DNA template. RNA products, including HDV ribozymes (Hepatitis-Delta-Ribozyme) and uncut RNA, were separated by preparative urea–polyacrylamide gel electrophoresis (PAGE). tRNA^Ile^ was then extracted from the gel, ethanol precipitated, and desalted using a PD10 column (Cytiva, Marlborough, Massachusetts). The RNA was concentrated using a SpeedVac (Thermo Fisher Scientific, Waltham, Massachusetts) and subsequently stored at –20°C.

### RNase T1 digestion of tRNA into oligonucleotides

For RNase T1 digestion, RNase T1 (Thermo Fisher Scientific, 1000 U/µl) was diluted in 25 mM Tris–HCl (pH 7.5) and 100 mM NaCl. Up to 1 µg of RNA was digested with RNase T1 at 37°C for 30 min in a total reaction volume of 50 µl, maintaining final concentrations of 25 mM Tris–HCl (pH 7.5) and 100 mM NaCl. For the IVT and biological samples, a final ratio of 10 U RNase T1 per µg of RNA was used.

### RNase 4 digestion of tRNA into oligonucleotides

The protocol was adapted from Wolf *et al*. [27]. RNA was initially denatured by adding 3 M urea (Carl Roth, Germany) to achieve a final concentration of 1 M. The sample was then incubated at 90°C for 10 min, followed by immediate transfer to and maintenance at 37°C. Subsequently, the cooled RNA solution was diluted three-fold in NEBuffer r1.1 (New England Biolabs) by adding twice the volume of 1.5× NEBuffer r1.1, which had been pre-warmed to 37°C. Human RNase 4 (New England Biolabs) was pre-diluted to the desired concentration, and 1 µl of the diluted enzyme was added to the RNA mixture. The reaction was incubated at 37°C for 1 h. For the IVT and biological samples, a final ratio of 20 U RNase 4 per µg of RNA was used.

### Colicin E5 digestion of tRNA into oligonucleotides

For colicin E5 digestion, 1 µg of *in vitro* transcribed tRNA was incubated with colicin E5 at a molar ratio of 4:1 (colicin E5:RNA) in a reaction buffer containing 25 mM Tris–HCl (pH 7.5) and 100 mM NaCl. The total reaction volume was adjusted to 50 µl with nuclease-free water. The reaction mixture was gently mixed and incubated at 37°C for 30 min.

### Polyacrylamide gel electrophoresis

Oligonucleotides were analyzed using 20% TBE–urea PAGE, which was prepared using ROTIPHORESE^®^ gel concentrate, buffer concentrate, and gel dilute from Carl Roth (Karlsruhe, Germany). Before sample loading, the gel was equilibrated by pre-run at 250 V for 30 min. Samples (20 pmol) were mixed 1:1 with 2× loading dye (NEB, Ipswich, MA, USA) and loaded into wells (20 µl per sample). A molecular ladder containing 10 pmol of oligonucleotides (5-, 8-, 10-, 20-, 30-, 40-mer, tRNA^Ile^, HDV) in 2× RNA loading dye was prepared and loaded. Electrophoresis was performed at 275 V for 60–90 min. The gel was stained for 10 min with Stains-All solution [0.65× TBE, 0.01% Stains-All (Thermo Fisher Scientific), 10% formamide, 25% isopropanol, 65% water], destained for 2 h in 1× TBE buffer containing 25% isopropanol, and imaged using a Bio-Rad ChemiDoc™ MP Imaging System.

### Sample preparation for Matrix-Assisted Laser Desorption/Ionization-Time Of Flight (MALDI-TOF) MS and nanoflow HILIC-MS

To prevent RNA over-digestion, samples were filtered immediately after RNase-digestion using Zymo-Spin filters (C1004-50, Zymo Research Europe GmbH, Freiburg, Germany) according to the manufacturer’s instructions. Following incubation, the reaction mixture (50 µl) was combined with 100 µl of Zymo oligo-binding buffer. Subsequently, 400 µl of absolute ethanol was added, and the resulting solution was mixed thoroughly. The entire mixture was loaded onto a Zymo-Spin column and centrifuged at 12 000 × *g* for 30 s. After discarding the flow-through, 750 µl of DNA wash buffer was added to the column, followed by a second centrifugation at 12 000 × *g* for 60 s. The Zymo-Spin column was then transferred to a clean microcentrifuge tube, and 6–10 µl of RNase-free water was carefully applied to the center of the column membrane. To elute the purified oligonucleotide fragments, the column was subjected to a final centrifugation at 12 000 × *g* for 60 s. The eluted RNA was stored at −20°C for further analyses by MS.

### MALDI-TOF mass spectrometry of oligonucleotides

A mixture of 3-hydroxypicolinic acid (3-HPA) and diammonium hydrogen citrate (DAC) was prepared as the MALDI matrix as follows. First a solution of 10 mg/ml DAC in H_2_O:acetonitrile (1:1; v:v) was prepared. Then 3-HPA was added to this solution until saturation. A defined volume was taken and mixed with an equal volume of 10 mg/ml DAC in H_2_O:acetonitrile (1:1; v:v) to receive a half-saturated solution of 3-HPA [45].

For each sample, 0.5 µl of this 3-HPA:DAC matrix were spotted onto an AnchorChip target (800 µm). The matrix solution was allowed to dry at room temperature. Then 0.5 µl of each sample (7.75 µM) was spotted on top of the dried matrix spot. The sample was allowed to dry and the spectra were acquired in positive ion mode with reflector on an ultrafleXtreme MALDI-TOF-TOF mass spectrometer (Bruker Daltonics, Bremen, Germany).

### Liquid chromatography (tandem) mass spectrometry-based oligonucleotide analysis

**Materials** used for LC development: Thermo RP column: Acclaim™ PepMap™ 100 C18 HPLC column (150 mm x 0.075 mm, 2 μm, 100 Å, cat# 164534), RP trap cartridge: PepMap™ Neo Trap Cartridge, (5 mm x 0.3 mm, 5 μm, 100 Å, cat# 174500), and Thermo amide: Accucore™ 150 Amide HILIC HPLC column (150 mm x 0.075 mm, 2.6 μm, 150 Å, cat# 16726-157569, Thermo Scientific Inc., Karlsruhe, Germany). HILIC stem trap cartridge: EXP2 0.33 µl stem trap (13.5 mm x 180 μm, 5 μm HALO PELL HILIC, 90 Å, P/N: 15-04001-ES, Optimize Technologies, Oregon, USA). Self-packed nano diol column was 150 mm x 0.075 mm with ReproSil-Pur 120 Diol, 3 μm, 120 Å, P/N: r13.d0.0001, Dr. Maisch HPLC, Ammerbuch, Germany.

**Sample preparation** was performed as outlined in the section above. For assessing the impact of 100% aqueous injection versus 20% aqueous RNA hydrolysate injection, samples were premixed with 80% acetonitrile prior to injection.

The LC-MS-based oligonucleotide analysis was performed on a UHPLC Ultimate™ 3000 RSLCnano system coupled with a Q Exactive Plus hybrid quadrupole-Orbitrap mass spectrometer (Thermo Fisher scientific, Germering, Germany). For the Q Exactive Plus measurements, a Pepsep sprayer (P/N PSS1, Bruker, Billerica, MA, USA) was paired with a Pepsep integrated liquid junction stainless steel emitter (P/N 1893525, 30 μm ID, Bruker, Billerica, MA, USA).

Samples were injected either via the “direct” or “pre-concentration” injection modes during method optimization using 80/20 (v/v) of 15 mM ammonium acetate (pH 5.5)/ACN as mobile phase A (MPA) and 20/80 (v/v) of 15 mM ammonium acetate (pH 5.5)/ACN as mobile phase B (MPB) [22]. Samples were pre-diluted in 80% ACN before injection. While the “pre-concentration” injection mode was conducted, samples were firstly trapped onto a EXP2 0.33 µl stem trap using MPB for 5 min at a 5 µl/min flow rate, unless otherwise mentioned. Afterward, the pre-concentrated samples on trap were reverse-eluted onto the nano analytical column depending on the experiment using a 0.3 µl/min flow rate at 30°C, unless otherwise stated. The LC gradients for different experiments are shown in the respective section. Blank injections (i.e. MilliQ water) were included between every sample run. UV absorption at 260 nm was recorded throughout the elution.

**Settings on the Q Exactive Plus**: Post-column analytes were ionized under 1.8 kV spray voltage in the negative ion mode at 250°C and analyzed using the “Full MS/data-dependent MS^2^ (dd-MS^2^)” method with a scan range of 500–2500 *m*/*z*. Resolution, AGC target, and maximum injection time for both MS and MS^2^ were set to be 70 000 (FWHM), 1e6, and 300 ms, respectively. The Top 15 most intense ions were selected for MS^2^ fragmentation in the higher-energy collisional dissociation cell using the normalized collision energy set to be 28 with 10 s dynamic exclusion period with *m*/*z* 1.7 isolation window width.

### NucleicAcidSearchEngine analysis

The LC-MS raw data files were converted to mzML format files using MSConvert (https://github.com/ProteoWizard/) and analyzed using the NucleicAcidSearchEngine (NASE) software (OpenMS ver. 3.0.0-pre-nightly-2023-03-09, https://openms.de/) for database matching [23, 46, 47].

Target/decoy database searches were performed with 5% false discovery rate cutoff. Mass tolerances for precursor and fragment ions were set to be 10 ppm each, while precursor charge states of −2 to −20 were included. Potential cation adducts, including sodium (Na^+^), potassium (K^+^), and ammonium (NH^4+^), were included in the search for their potential contribution to the shift of precursor *m*/*z* values. Precursor isotopes of −1, 0, 1, 2, and 3 were included, while all possible fragment ion types, including a-B, a, b, c, d, w, x, y, and z ions, were selected in the searches. The detailed configuration used for all our data analysis procedures is specified in Supplementary Fig. S24, where the corresponding nuclease must be chosen in the option “enzyme.” The NASE analysis of synthetic ribonucleotides and poly-dT oligonucleotides were manually verified in Skyline software (ver. 22.2.0.351, University of Washington, Seattle, WA, USA) using self-prepared transition list, including retention time, isotopic correlation, and precursor mass tolerance [48].

### Use of an AI language model

We used an AI assistant (GPT-5 Thinking) to support manuscript preparation in well-defined, author-directed tasks. Specifically, we prompted the model to (i) extract the transcribed FLuc mRNA sequence from the provided PDF and generate a FASTA file and (ii) language editing for clarity, logical flow, structure, concision, terminology harmonization, and grammar. All AI-generated outputs were reviewed, edited, and verified by the authors; data analysis decisions, figures, and text content remain the responsibility of the authors.

## Results

### Comparison of RNase activity

**Figure 1.**
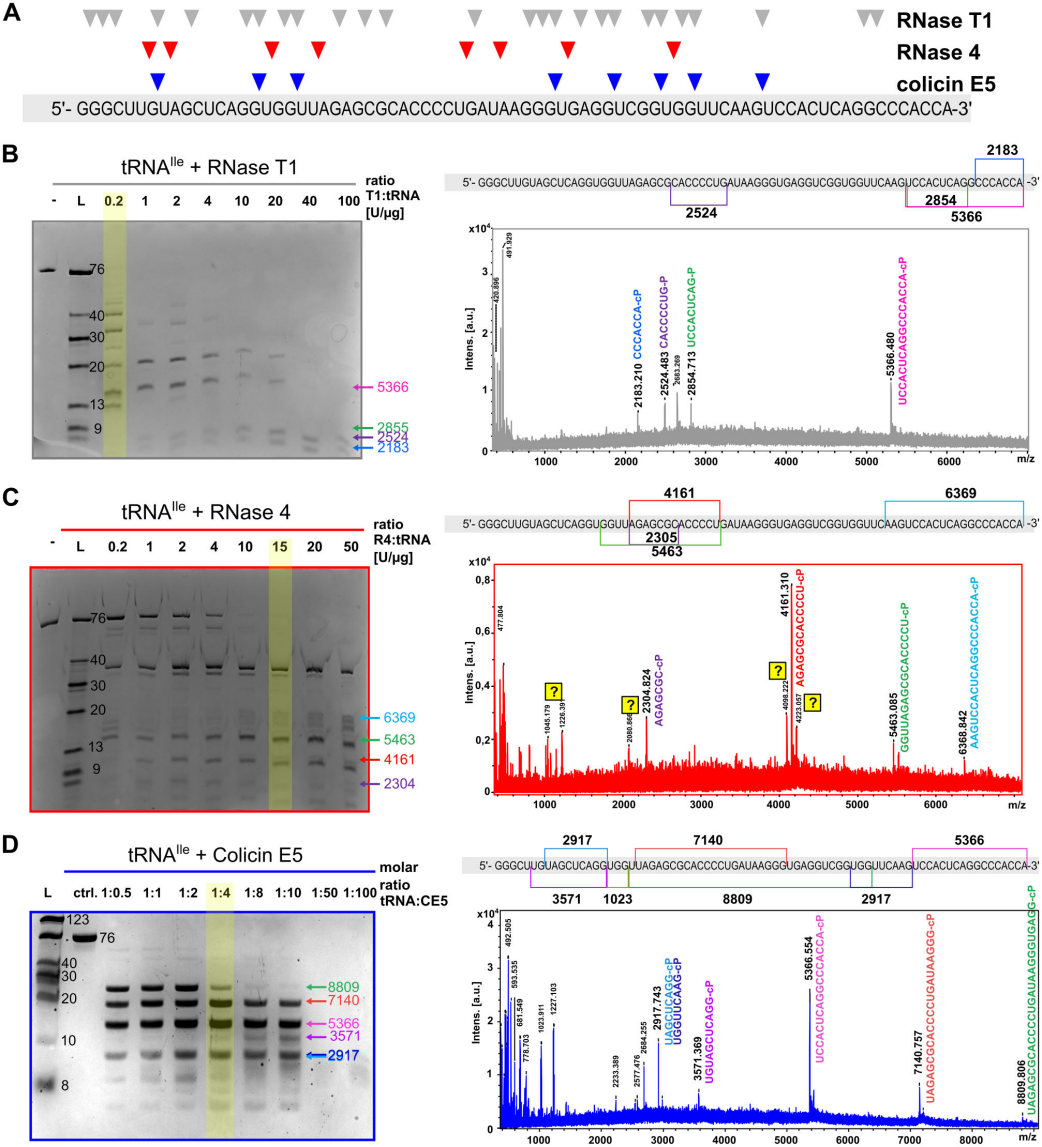
Substrate specificity of common nucleases for partial RNA hydrolysis. (**A**) sequence of an unmodified tRNA (*E. c*. tRNA^Ile^_GAU_) and the expected cleavage sites of three common RNases. RNase T1—gray, RNase 4—red, colicin E5—blue. (**B**–**D**) polyacrylamide gel of *E. c*. tRNA^Ile^_GAU_ treated in different ratios with the three RNases and the corresponding MALDI-MS spectra. Assigned fragments and cleavage sites are added to the MS spectrum and in the sequence above. Digestion conditions: 37°C, 30 min for RNase T1 and colicin E5 and 60 min for RNase 4. Arrows in the gel give the [M+H]^+^ of the indicated bands. cP = 3′ cyclic phosphate and P = 3′ linear phosphate.

To verify the activity of the purchased RNases T1 and 4 as well as our purified colicin E5, we first performed a cleavage assay using an *in vitro* transcribed tRNA^Ile^ as a benchmark RNA for the following experiments. The sequence of tRNA^Ile^ contains several G’s, UG/UA, and GU motifs, allowing us to follow potential cleavage events at different sites for RNase T1, RNase 4, and colicin E5 (Fig. 1A). One microgram of RNA was incubated with increasing amounts of each RNase according to the respective RNase’s standard protocol and the RNA was analyzed on a 20% denaturing acrylamide gel. As expected, all enzymes were active based on the disappearance of the full-length tRNA band and the appearance of product bands below the tRNA band (Fig. 1B–D). Furthermore, with lower amounts of enzyme we observed longer fragments, potentially due to missed cleavages. We then subjected all samples to MALDI-TOF MS analysis and determined the mass of the resulting fragments. An exemplary spectrum of an intermediate enzyme concentration is found for each RNase in Fig. 1B–D and for all other concentrations in Supplementary Figs S2–S4. With rising enzyme:RNA ratio, the MALDI spectra changed toward lower *m*/*z* values consistent with a reduced number of missed cleavages. For RNase T1, we confirmed that 3′-termini formation depends on RNase concentration. At low enzyme concentrations, cleavage predominantly yields 2′,3′-cyclic phosphate intermediates, whereas higher enzyme concentrations result in the accumulation of 3′-phosphate ends [49].

For RNase 4, we also observe changes in the MS spectra in dependence of the studied RNase concentration. Unlike with RNase T1, we do not see smaller fragments with rising enzyme concentration but rather different fragments under the different conditions. This, and the fact that we could not assign all fragments according to the expected U|A and U|G cleavage site, indicates that the substrate specificity of RNase 4 is more variable. At higher enzyme-to-substrate ratios, we additionally detected an increased frequency of C|A cleavage sites, consistent with previous findings [19]. This highlights the importance of applying moderate enzyme-to-substrate ratios when specific cleavage patterns are desired. Notably, RNase 4 predominantly generates 2′,3′-cyclic phosphate ends. On the gel we noticed a long fragment around 35 nt, which is probably the result of a missed cleavage in a double-stranded stretch of the tRNA substrate. This fragment, however, was not detected in the MALDI-TOF spectrum. In our MALDI-MS setup, no fragments exceeding 25 nt were detected, potentially due to a combination of decreased ionization efficiency for larger, highly charged oligonucleotides and increased cation adduct formation, which distributes the signal intensity across multiple peaks, as previously described [50].

For colicin E5, all MALDI spectra were similar and did not depend on the chosen concentration of enzyme. Most signals in the MALDI spectrum could be assigned to the expected G|U cleavage site and most fragment signals were with a 2′,3′-cyclic phosphate group, rather than a 3′-phosphate.

All in all, our results show that all three enzymes can produce oligonucleotides within a suitable length range (5–40 nt) for downstream MS analysis of native RNA. However, all three RNases exhibited substantial cleavage variability, including 3′-termini or cleavage site specificity, complicating downstream data analysis. To minimize this effect, we therefore systematically assessed the influence of the reaction conditions on digestion product variability.

### Variability of reaction products is only mildly influenced by the reaction conditions

To further assess the impact of the enzymatic digestion conditions on the resulting oligonucleotide products, we defined “starting” parameters for each nuclease (Supplementary Table S4) and varied incubation time, temperature, buffer composition (including magnesium ions), and pH. Oligonucleotide length was assessed by gel electrophoresis. As shown in Supplementary Fig. S5, reaction products generated by colicin E5 remained highly similar across all tested conditions. For RNase T1 and RNase 4, temperature variation had only a moderate effect on digestion pattern. Both enzymes were active even on ice, with RNase T1 and RNase 4 showing maximum activity at around 37°C and 50°C, respectively. Under those conditions, no RNase T1 fragments longer than 20 nt and no intact tRNA after RNase 4 digestion were detectable. At 50°C, shorter fragments were observed. Increasing the temperature to 75°C, however, inhibited enzymatic activity, leading to the appearance of longer fragments. Yet, residual cleavage activity was still observed, confirming that heat inactivation at 75°C is insufficient for complete enzyme inactivation.

For all three nucleases, rapid reaction kinetics were observed under chosen experimental conditions. Specifically, RNase T1 and RNase 4 generated distinct and reproducible cleavage products within a 5-min incubation period. Prolonged incubation led to the generation of shorter fragments.

We further compared the digestion efficiency under different buffer conditions, including NaCl/Tris at pH 7.8, NEBuffer™ r1.1 (supplied with RNase 4), and ammonium acetate at pH 5.5 and 7.0 (Supplementary Fig. S5). RNase T1 produced similar cleavage products under all buffer conditions, whereas RNase 4 showed buffer-dependent differences in fragment patterns, particularly for fragments <30 nt. RNase T1 was active at both pH 5.5 and 7.0, while RNase 4 retained higher activity at pH 7.0. Without any salt in the digestion buffer, RNase 4 showed no activity, and RNase T1 exhibited only minimal activity.

Our results indicate that RNase T1 and RNase 4 produce the most variable digestion patterns, while hydrolysates of colicin E5 are homogenous in length and largely insensitive to the tested conditions. However, it is important to note that the influence of individual parameters is interdependent; thus, our conclusions are valid only for the tested parameter combinations.

### High resolution MS analysis of RNA hydrolysates using nanoflow HILIC

To achieve the highest possible sensitivity, we reduced the flow rate to nanoflow, which is a format that has never been established for HILIC-based oligonucleotide analysis.

**Figure 2.**
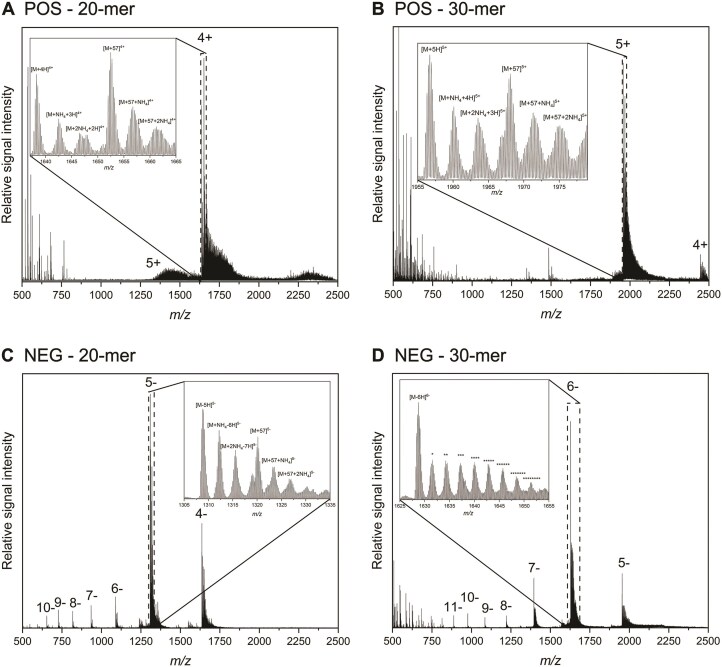
MS^1^ spectra comparison of 20-mer (left) (**A, C**) and 30-mer (right) (**B, D**) (synthetic ribonucleotides) between positive ion mode (POS—upper spectra) and negative ion mode (NEG—lower spectra). Charge states of some intense peaks in each spectrum were indicated. A zoom-in spectrum of the most abundant charge states was inserted in each spectrum. The numbers of asterisk (*) indicate the numbers of NH_4_ adducts, e.g. “***” at “6-” indicates [M+3NH_4_-9H]^6−^.

For method development and optimization, we used synthetic RNA and DNA oligonucleotides (sequences in Supplementary Table S3). Figure 2 shows the mass spectra of directly injected 20-mer and 30-mer RNA using a HILIC set-up and HILIC-suitable mobile phases. Retention times are 38, 55, and 58 min for the 5-, 20-, and 30-mer, respectively (Supplementary Fig. S6, including an additional 5-mer). Oligonucleotides are commonly ionized in negative mode due to the negatively charged phosphodiester backbone [16]. Yet, at acidic pH, nucleobases are easily charged and thus analysis in positive mode is also possible [40]. We find that both ionization modes are suitable for analysis of oligonucleotides from 5–30 nt, although the mass spectra differ substantially.

In positive ion mode, all tested oligonucleotides have a dominating charge state in their corresponding mass spectra with signal abundance >95% (Fig. 2, top panel). This contrasts with negative ion mode, where we also find one dominating charge state, but for the longer oligonucleotides additional charge states occur that account for at least 40% of signal abundance (Fig. 2, bottom panel). In positive ion mode, the signal of the oligonucleotides is not split into multiple peaks, which improves sensitivity compared to negative ionization. Positive ion mode is therefore well suited for sensitive, targeted MS1-based oligonucleotide analysis and produces less complex spectra. Negative ionization, in contrast, provides higher precursor charge states at MS1 level, which benefits subsequent MS/MS fragmentation. Oligonucleotides fragment along the phosphodiester and the McLuckey nomenclature defines the possible a, b, c, and d-ions (5′-end) and w, x, y, and z-ion (3′-end) (Supplementary Fig. S7) [51]. The detection of a high number of informative fragment ions is needed for confident sequence annotation and is supported by higher precursor charge states and more balanced charge distribution during MS/MS. Thus, meaningful MS/MS spectra with a high sequence coverage are preferentially observed in negative ion mode (Supplementary Fig. S8). Often, MS adducts can interfere with oligonucleotide analysis, particularly for (relative) quantification because the MS signal will be distributed across protonated precursor and potential adduct species. Common adducts include alkali ions such as sodium (Na^+^  *m*/*z* = 23) or potassium (*K*^+^  *m*/*z* = 39) and ammonium ion (NH_4_^+^  *m*/*z* = 18). The abundance of alkali ions can be reduced by properly cleaning borosilicate glass [52] and avoiding sodium containing buffers. This strongly exemplifies the importance of using a clean system with minimal salt load for successful and reproducible oligonucleotide analysis. A zoomed view of the most abundant peak in each spectrum reveals a similar abundance of ammonia adducts in both positive and negative ion modes. Further, an adduct with *m*/*z* = 57 is observed, which we cannot assign at the moment.

All in all, we conclude that positive ion mode is advantageous for highly sensitive targeted analysis, whereas negative ion mode is preferable for MS/MS-based oligonucleotide sequencing.

### HILIC trap columns enable sample loading for nanoflow HILIC oligonucleotide analysis

Nanoflow chromatography often benefits from using a trap column as it improves the peak intensity and sensitivity, especially if high loading volumes are needed. To test the applicability of the trap system in the nanoflow HILIC set-up, we installed a HILIC stem trap and loaded synthetic oligonucleotides for 5 min at a flow rate of 10 µl/min. The sample was then separated at 250 nl/min using either no analytical column, a reverse phase column, or a commercially available HILIC-amide column. We found that the HILIC trap column was able to retain the oligonucleotides, and substantial peaks were observed upon elution into the nanoflow path. As expected, the oligonucleotides were not separated when no columns or an RP column were used (Supplementary Fig. S9A or B). In contrast, the oligonucleotides were well separated on the HILIC-amide analytical column (Supplementary Fig. S9C). To further evaluate the impact of the HILIC trap on the subsequent HILIC separation, the oligonucleotides were directly injected (without a trap column and loading step). In direct injection (Supplementary Fig. S9D), both oligonucleotides elute earlier but showed similar peak shapes. These results indicate that the HILIC-amide analytical column has a stronger retention than the HILIC trap column, which is important for successful analyte trapping and thus preconcentration. Based on these observations, we recommend using the trap set-up if injection volume exceeds 1 µl. For concentrated samples with a 1 µl injection volume, direct injection can be used and is preferable as a trap set-up is more prone to leaks and system malfunction compared to direct injection.

### High water content destabilized electrospray and MS detection of oligonucleotides

Since oligonucleotides eluted earlier under the direct injection setup while maintaining acceptable resolution (Supplementary Fig. S9) and because bypassing the trap column simplifies both workflow and troubleshooting, we chose direct injection for biological samples. However, in nanoflow direct injection, the injection volume (1 µl) exceeds the column dead volume (0.66 µl for a 15 cm × 75 μm column), making the system highly sensitive to the injection solvent. To confirm the necessity of matching HILIC initial conditions, we evaluated the effects of aqueous injections using a synthetic 8-mer. Injecting the sample in 100% water resulted in sample breakthrough, severe electrospray instability, and a distinct pressure increase from ∼60 bar to ∼110 bar (Supplementary Fig. S10A and B). In contrast, injecting sample in 100% MPB (80% acetonitrile with buffer) ensured robust retention, stable system pressure, and reproducible chromatographic performance (Supplementary Fig. S10C and D). Consequently, to prevent both sample breakthrough and the solvent-induced instabilities described above, all subsequent sample injections were strictly performed in 100% MPB.

Beyond its impact on sample introduction in direct injection mode, high water content also poses a critical challenge during MS detection. During oligonucleotide analysis in both direct injection and trap mode, we noticed an MS signal loss when the water content of the mobile phase composition exceeded 50%. Using the commercial HILIC-amide column, long oligonucleotides were strongly retained and eluted at high water content, where their signal became unstable, as shown for the 5-, 20-, and 30-mer in Supplementary Fig. S11. We found that the electrospray was more stable at higher radio-frequency voltage (RF) values at the ion funnel and for some oligonucleotides the signal could be recovered by using an RF of 90 (Supplementary Fig. S11). However, the electrospray instability persisted. If a second nanoflow pump is available, the content of organic solvent can be increased after the chromatographic separation and prior to the ion source. The set-up is shown in Supplementary Fig. S12, and its impact on the MS signal intensity is given in Supplementary Fig. S11C–F. With this post-column infusion setup, the total ion count (TIC) was stable for all analyzed oligonucleotides, even at lower RF values of 50. In addition, the TIC signal intensity was similar or even higher than that observed without infusion. Detailed analysis of MS1 charge state distributions (Supplementary Fig. S13) and MS/MS fragmentation (Supplementary Fig. S14) revealed various advantages of the infusion set-up, including stable electrospray, high sensitivity, and good sequence coverage.

### Reduced retention on HILIC-diol column enables stable electrospray

**Figure 3.**
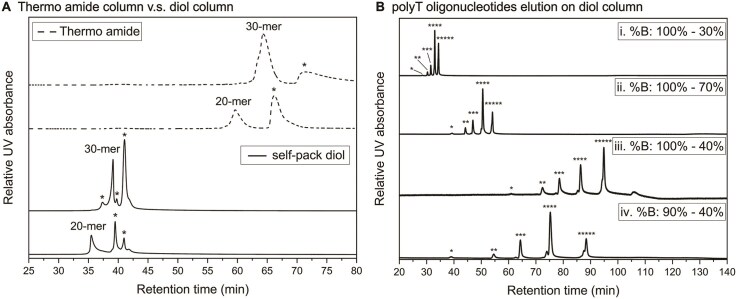
Separation of oligonucleotides on nanoflow HILIC using amide or diol chemistries. (**A**) UV chromatograms of a 20-mer and a 30-mer (synthetic ribonucleotides) eluted on the commercial HILIC-amide column (shown on top in dashed lines) and the self-packed diol column (shown in the bottom in solid lines). Impurities present in these samples were marked by the asterisk (*) signs. Note: The LC gradient used for the self-pack diol column (0.30 µl/min) was marginally (i.e. 9 min) longer than the HILIC-amide column (0.25 µl/min). (**B**) Separation of dT-only containing oligonucleotides with different gradients (i, ii, iii, and vi, which were specified in the supplementary method) on the self-packed diol column. “*”, “**”, “***”, “****”, and “*****” indicated samples “dT10,” “dT15,” “dT20,” “dT30,” and “dT50,” respectively.

Another strategy to generate a continuous and stable spray is to use another column chemistry with lower oligonucleotide retention. Because nanoflow HILIC columns are not commonly available, we tested different HILIC materials in self-packed columns. Among the materials tested, the diol chemistry (dihydropropane groups chemically bonded to porous silica particles) showed the best usability because it provided sufficient oligonucleotide separation while reducing retention. A comparison of the commercial HILIC-amide and the self-packed HILIC-diol column toward the separation of a 20-mer and 30-mer is shown in Fig. 3A. Oligonucleotides elute earlier on the diol column and thus at higher organic phase content. Accordingly, we did not observe a loss of electrospray stability with this column. Regarding the separation performance, we analyzed a mixture of synthetic deoxythymidine oligonucleotides (dT) containing 10, 15, 20, 30, or 50 dTs. The separation of these oligonucleotides was influenced by gradient steepness as seen in Fig. 3B. Even with a steep gradient, a resolution above 5 is achieved for all peak pairs (Supplementary Fig. S15), all oligonucleotides elute within 40 min and a full MS/MS coverage of the analyzed poly-dT oligonucleotides (Supplementary Fig. S16) was obtained.

Regarding sensitivity, ~0.1 ng/nucleotide, corresponding to 2 ng of a 20-nt oligonucleotide, was sufficient for its detection and successful sequence mapping using the self-packed HILIC-diol nanoflow set-up (Supplementary Table S5).

### Analysis of partial RNA hydrolysates using nano-HILIC-HRMS

**Figure 4.**
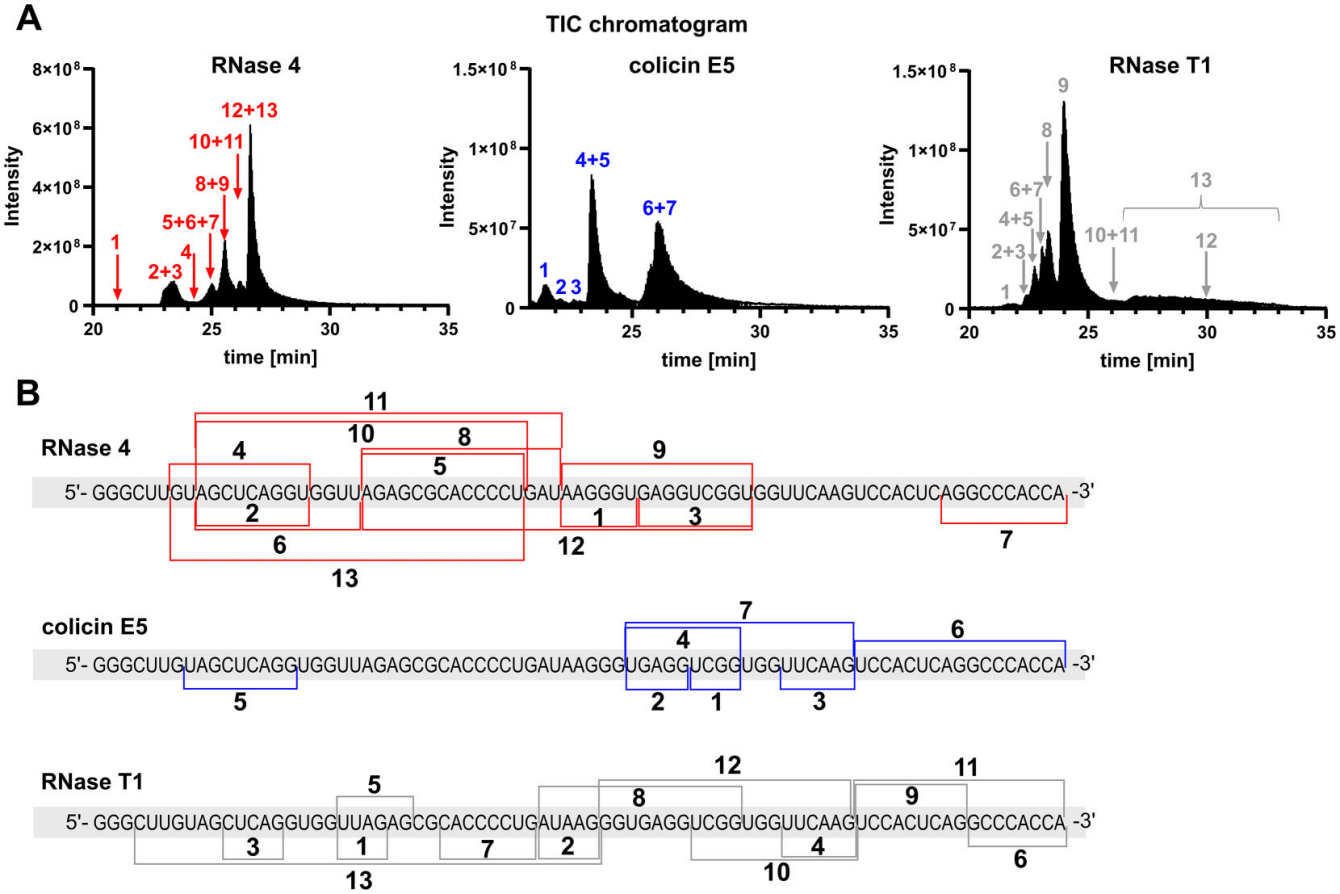
Comparison of RNase T1, RNase 4, and colicin E5 digested unmodified RNA (E. c. tRNA^Ile^_GAU_) in the developed nanoflow-HRMS set-up. (**A**) TIC chromatograms with arrows indicating RT of found fragments. (**B**) Assigned fragments and cleavage sites in the sequence of E. c. tRNA^Ile^_GAU_. Red: RNase 4; blue: colicin E5; gray: RNase T1.

After successful detection of synthetic oligonucleotides in our system, we digested unmodified tRNA^Ile^ with RNase T1, RNase 4, or colicin E5. For removal of residual salt and protein, we used the same sample preparation workflow as for our MALDI MS analysis based on Zymo spin column clean up. This method was suitable for robust HILIC nanoflow chromatography. We injected 50 ng of hydrolyzed tRNA onto the column using direct injection (1 µl) and the resulting total ion chromatograms are shown in Fig. 4. For all three nucleases, cleavage products within a suitable length range (5–40 nt) were detected. The oligonucleotides previously detected by MALDI-MS were readily detectable at high resolution and a mass accuracy below 10 ppm. MS/MS spectra were acquired for these peaks and the observed fragmentation matched the *in silico* expected fragments predicted by the Mongo Oligo Mass Calculator v2.07 [53].

### NASE-based fragment assignment requires defined 3′-end chemistry after hydrolysis

With the oligonucleotide-MS method successfully developed, we utilized it to study the substrate specificity of the three RNases in more detail. For 3′ termini determination, we did not utilize a phosphatase at this stage. Instead, we used NASE and its search function for the respective RNase specific cleavage and “unspecific cleavage.” Unspecific cleavage refers to a bioinformatic search strategy within NASE. This search mode is a non-restrictive *in silico* digestion where the software considers every phosphodiester bond as a potential cleavage site. It allows unbiased identification of potential off-target cleavage events.

**Figure 5.**
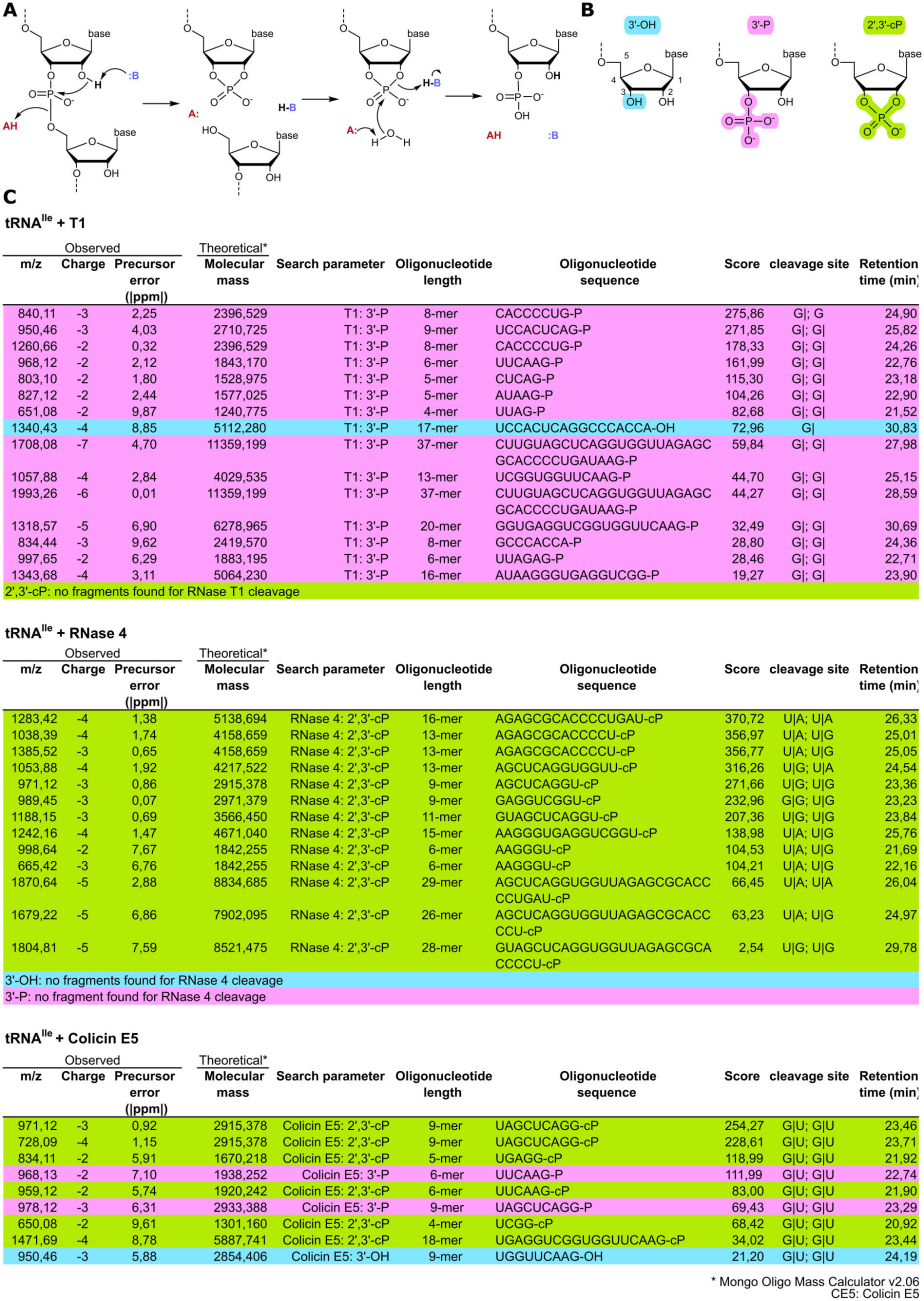
Comparison of RNase 4, colicin E5, and RNase T1 digested unmodified RNA (*E. c*. tRNA^Ile^_GAU_) using updated NASE. (**A**) General Ribonuclease-mechanism with AH indicating an acidic amino acid and B indicating a basic amino acid. (**B**) Chemical structures of potential 3′ ends of RNase hydrolysis. (**C**) Table of identified fragments of tRNA^Ile^_GAU_ for RNase T1, RNase 4, and colicin E5. cP = 3′ cyclic phosphate, P = 3′ linear phosphate, and OH = 3′ hydroxyl. In the column “cleavage site,” the 5′- and 3′-cleavage sites are separated by semicolon (;).

Problematically, when we started analysing our data using the RNase specific cleavage, not all oligonucleotides detected by MALDI-MS could be assigned by NASE. From our previous analysis by MALDI-MS (Fig. 1) and manual data analysis (Fig. 4) we observed that the 3′-end of the oligonucleotides can carry a 2′,3′-cyclic phosphate, a 3′-phosphate, or a 3′-hydroxyl group (Fig. 5A and B). We learned that the 3′-end selection is crucial for correct fragment assignment and therefore, NASE was modified to allow selection of different 3′-chemistries.

With these new developments in NASE, we moved forward with analysis of the oligonucleotides produced by the three nucleases. RNase T1 strictly targets G|N sites and primarily produces fragments with 3′-phosphate termini under the applied conditions (Fig. 5C). Although complete hydrolysis with RNase T1 generates fewer unique products (1–9 nt), partial digests with reduced enzyme-to-substrate ratios and limited incubation times produced longer fragment of up to 39 nt. This approach provided the highest sequence coverage of all nucleases tested (96.1%), with no fragments detected in the non-specific search mode (Supplementary Fig. S17).

We then moved on to analysis of RNase 4. Using the RNase 4 specific search function in NASE, oligonucleotides emerging from the expected U|A and U|G cleavage were readily detected if combined with 2′,3′-cyclic phosphate termini in the search. The fragment lengths ranged from 6 to 29 nucleotides, with sequences such as AGAGCGCACCCCUGAU-cP identified with high confidence.

In contrast to RNase T1, RNase 4 yielded a narrower fragment distribution, with partial redundancy observed in the middle regions of the tRNA^Ile^ sequence. We searched for 2′,3′-cyclic phosphate, 3′-phosphate, and 3′-OH ends in NASE and could only identify 2′,3′-cyclic phosphate products, which aligns with the reported RNase 4 activity at low enzyme concentrations [19]. Fragment overlap was most pronounced in uridine-rich loop domains and adjacent stem regions. For example, fragments such as AGAGCGCACCCUGAU-cP (16-mer) and AGAGCGCACCCCU-cP (13-mer) covered overlapping sequence regions but differed in cleavage end points, providing useful redundancy for sequence reconstruction.

We next performed “unspecific cleavage” analysis with NASE to determine potential off-target cleavages. As exemplified for RNase 4 products AGCUC-cP and AGGU-cP, we found an off-target activity at the C|A motif, as reported [19]. This may indicate either an expanded substrate tolerance or minor contaminating activities. Other off-target fragments were predominantly 4–10 nucleotides long and consistently carried 2′,3′-cyclic phosphate termini, supporting the enzyme’s overall mechanistic consistency. The ability of RNase 4 to cleave at both typical and unexpected sites resulted in a sequence coverage of 72.7%, of which ∼13% originated from C|A cleavage (Supplementary Fig. S18). These results support the suitability of RNase 4 for sequence mapping of highly purified RNAs, where limited off-target cleavage is a useful feature to gain maximum sequence coverage.

Colicin E5 was also assessed using the G|U-specific cleavage function of NASE. In this targeted search, a high number of cleavage products was detected, with abundant fragments spanning 4–18 nucleotides and carrying both 2′,3′-cyclic phosphate and 3′-phosphate ends. We found fragments that covered large regions of sequence and often overlapped by one or two nucleotides, a pattern that reflects highly redundant sequence coverage. This overlap was particularly noticeable in U- and G-rich regions, where multiple fragments’ start- and stop- positions created a pattern of quasi-continuous overlap.

Next, we searched for unspecific cleavage sites and found that colicin E5 generated various oligonucleotides outside the expected G|U motif. Detailed inspection revealed that these non-specificities were not fully random but followed sequence-dependent substrate recognition, suggesting a broader and potentially context-dependent substrate recognition. Cleavage was observed close to GU sites and especially in the context

G|G(G)U and GU|C indicating that colicin E5 can shift by 1–2 nucleotides. Furthermore, we observed occurrence of A|A, C|A, C|U, U|C, C|C, G|C, A|C, and U|U, which either indicates a broad substrate tolerance or a contamination with an unknown RNase. Those unspecific products have high scores in NASE and contributed 26% of the total sequence coverage, which reached 83.1% overall (Supplementary Fig. S18).

### RNase 4 and colicin E5 accept major RNA modifications within their substrate sites

**Figure 6.**
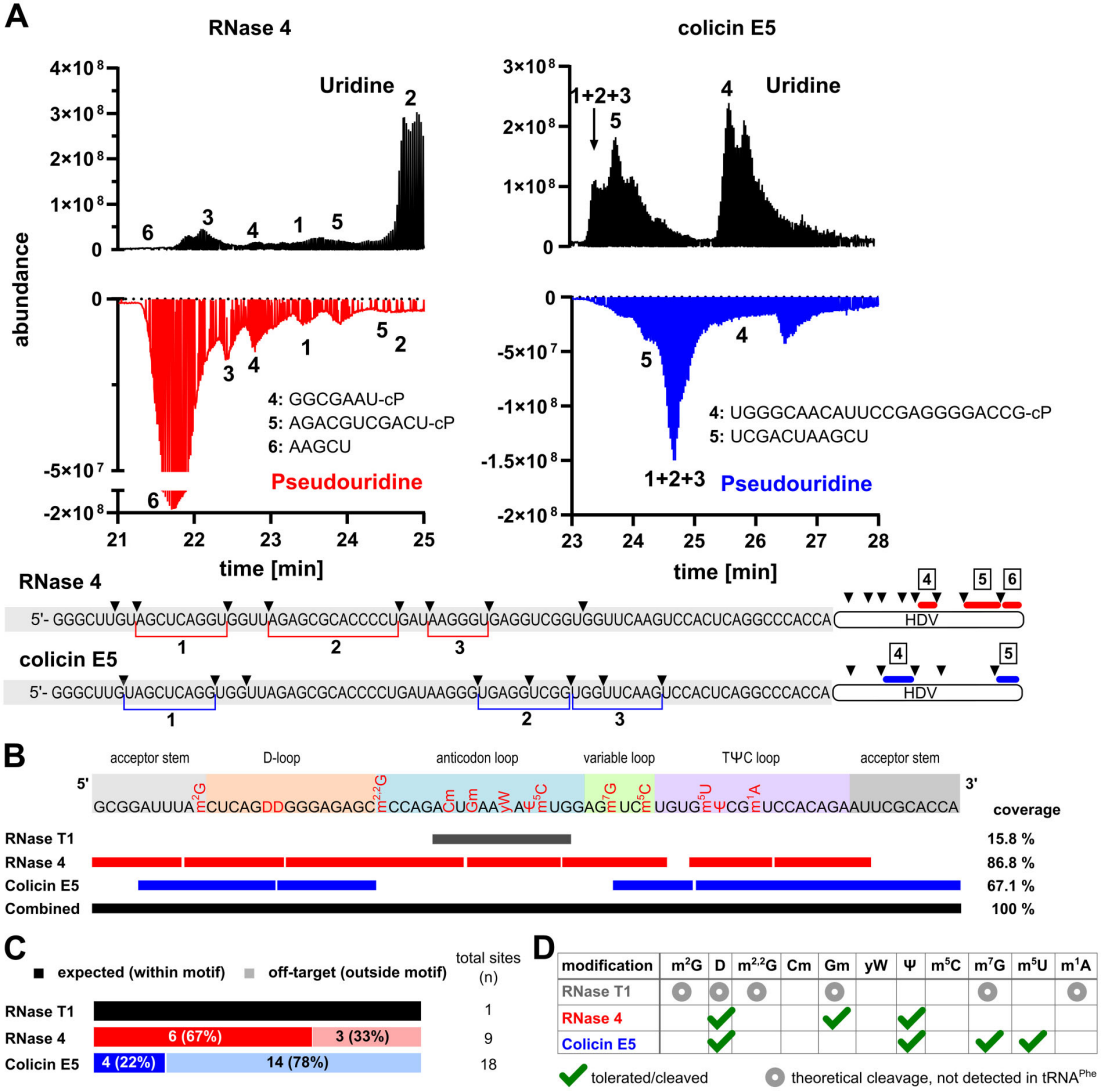
Application of the developed oligonucleotide-MS method with three different RNases to modified RNA substrates. (**A**) Analysis of an *in vitro*–transcribed (IVT) *E. c*. tRNA^Ile^_GAU_-HDV (Hepatitis-Delta-Virus ribozyme), prepared once with UTP (uridine triphosphate) and once with ΨTP (pseudouridine triphosphate), after digestion with RNase 4 (left) or colicin E5 (right). Top: TIC chromatograms of digested UTP-IVT samples; bottom: TIC chromatograms of ΨTP–IVT samples. Fragments identified by NASE are annotated in the sequence below. The numbered peaks in the chromatograms correspond to the labeled fragments. Red: RNase 4; blue: colicin E5. cP = 3′ cyclic phosphate. (**B**) Cleavage patterns of RNase T1, RNase 4, and colicin E5 mapped onto *S. c*. tRNA^Phe^_GmAA_. Horizontal bars indicate experimentally observed fragments positioned along the sequence. (**C**) Comparison of observed cleavage sites with expected motif specificity. Cleavage events were classified as on-target (within the canonical recognition motif) or off-target (outside the motif). (**D**) Influence of nucleotide modifications on RNase cleavage. Theoretical cleavage sites based on sequence motifs are compared to experimentally detected fragments. Check marks indicate observed cleavage, whereas gray circles denote expected but undetected cleavage sites. The identified RNA modifications are abbreviated as follows: D (dihydrouridine), m^2^G (N^2^-methylguanosine), m^2,2^G (N^2^,N^2^-dimethylguanosine), Cm (2'-O-methylcytidine), Gm (2'-O-methylguanosine), yW (wybutosine), Ψ (pseudouridine), m^5^C (5-methylcytidine), m^7^G (7-methylguanosine), m^5^U (5-methyluridine), m^1^A (1-methyladenosine).

Native RNA contains various modified nucleotides. Both RNase 4 and colicin E5 cleave in a uridine-containing dinucleotide context, which prompts the question, how the RNases handle the dominant RNA modification pseudouridine (Ψ) within their recognition sequence. To approach this question, tRNA^Ile^_GAU_ was *in vitro* transcribed in the presence of ΨTP without UTP. As a result, no uridine was in the RNA and all sites contained Ψ, which was confirmed by nucleoside MS (Supplementary Fig. S19). In the resulting RNA, the HDV ribozyme (Hepatitis-Delta-Virus self-cleaving ribozyme) was unable to release the full lengths tRNA. Instead, a longer RNA containing the HDV sequence was observed and purified for further analysis. The fully Ψ-modified RNA and a mixture of regular U-containing tRNA^Ile^ plus cleaved HDV RNA were incubated with RNase 4 and colicin E5. The resulting fragments were injected into the nanoflow-HILIC-HRMS system, and the TIC chromatogram is displayed in Fig. 6A. Both RNases were active and U- and Ψ-containing fragments showed identical MS1 and MS/MS features, supporting the identical cleavage positions. Ψ-containing fragments eluted marginally later than the corresponding U-containing fragments, which is in accordance with expectation due to Ψ’s higher hydrophilicity. From this data we conclude that RNase 4 and colicin E5 recognize both U and Ψ within their substrate sites. Due to Ψ being mass silent, we could not directly detect Ψ in these fragments, but its presence was confirmed by the production process. For direct detection of Ψ by MS, we recommend chemical derivatization [54–56].

To assess how RNase specificity translates into fragmentation patterns in a native RNA context, cleavage products from RNase T1, RNase 4, and Colicin E5 were mapped onto tRNA^Phe^ from *Saccharomyces cerevisiae* (Fig. 6B). In our hand, RNase T1 produced only one detectable fragment, while previous studies described the formation of several fragments [39]. Thus, our fragment map for RNase T1 is likely incomplete. In contrast, RNase 4 and Colicin E5 generated broader and partially overlapping fragment sets, resulting in substantially increased coverage. Combining all three enzymes enabled complete sequence coverage across the tRNA. We next compared observed cleavage sites to the expected motif preferences of each enzyme (Fig. 6C). RNase T1 cleavage was confined to its canonical recognition sites. In contrast, RNase 4 and colicin E5 exhibited extensive off-target activity, with a considerable fraction of cleavage events occurring outside their predicted motifs (Supplementary Fig. S20).

To determine whether RNA modifications contribute to these deviations, theoretical cleavage sites were compared to experimentally detected fragments (Fig. 6D). For RNase 4, we found successful cleavage for DΙG and UΙGm, while colicin E5 was active for GΙD, m^7^GΙU, GΙm^5^U, and the off-target site CΙm^2,2^G. We conclude that both RNases have a low stringency and may accept selected U- and G-modifications under tested conditions in this study. Systematic assessment should be the goal of future analysis, albeit the generation of synthetic, modified RNAs remains a major challenge in the field.

### Combining RNases is pre-requisite for high sequence coverage

**Figure 7.**
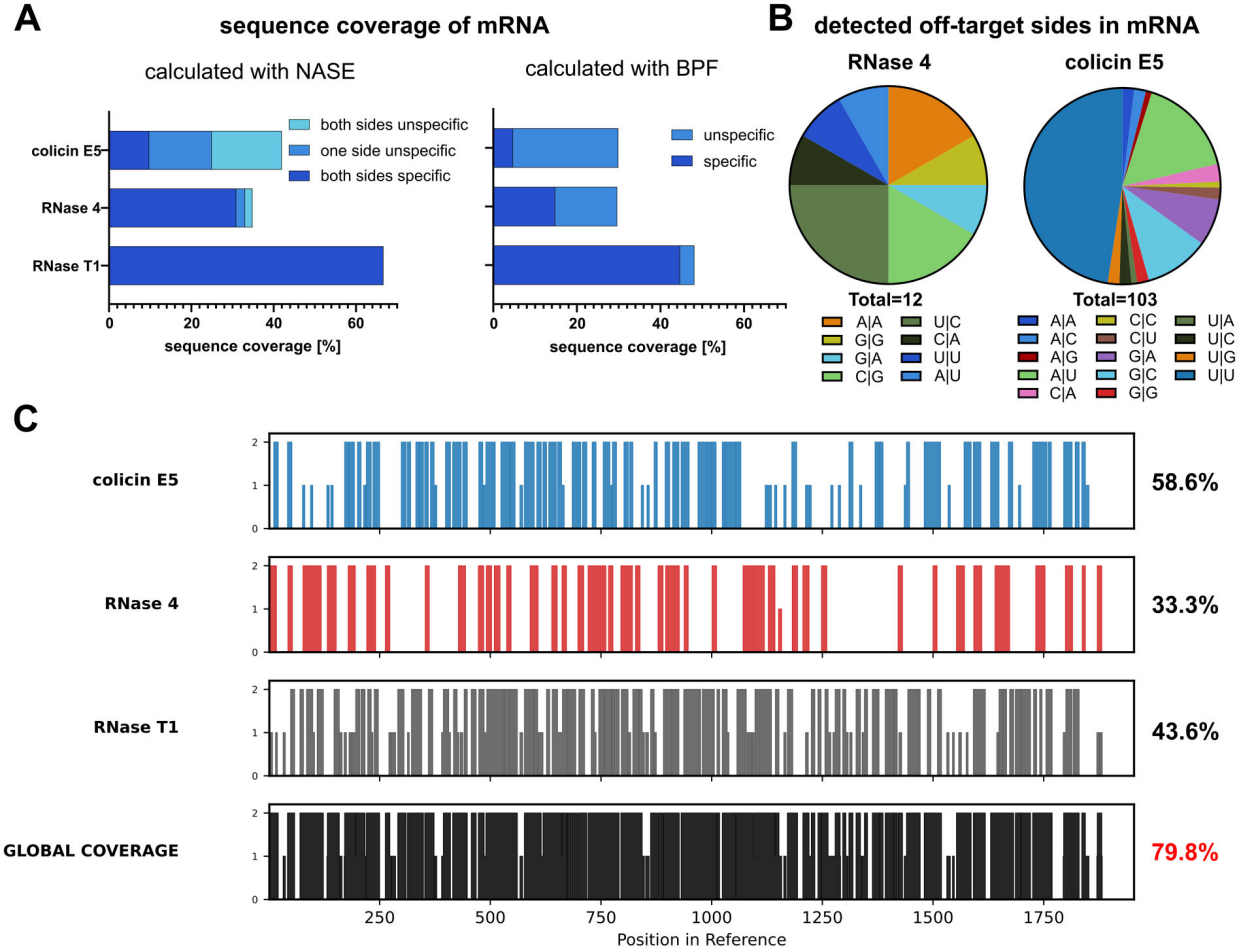
Analysis of FLuc mRNA which is digested with RNase 4, RNase T1 and colicin E5. (**A**) Sequence coverage obtained with each RNase, calculated with NASE and BPF (Thermo Fisher BioPharma Finder). Dark blue (left): fragments with both termini as expected specific cleavage sites; sky blue (middle): one terminus specific and the other unspecific; light blue (right): both termini unspecific. (**B**) Distribution of detected off-target cleavage sites in the mRNA for RNase 4 and colicin E5. No off-target sites were detected for RNase T1. (**C**) Sequence coverage maps obtained from individual digestions, calculated with NASE. Global coverage shows combined coverage from all three nucleases.

To assess how the observed RNase-specific cleavage behavior translates to complex RNA substrates, we next analyzed a long mRNA (FLuc) and evaluated sequence coverage and fragment uniqueness across nucleases. As the FLuc mRNA used in this study does not contain extensive natural RNA modifications, this analysis primarily reflects sequence-dependent cleavage behavior. For each RNase, 250-400 ng of mRNA hydrolysate were directly injected and separated on the described nanoflow-HILIC-MS set-up. The length of all detected oligonucleotides was plotted over the retention time (Supplementary Fig. S21). Similar to RP–IP methods, longer oligonucleotides are retained stronger and elute at later retention times. For partial RNase T1 digestion, a 58.6% sequence coverage was determined using NASE and 38.7% using BioPharmaFinder (BPF, proprietary software Thermo Fisher). As expected, no off-target cleavage was detected for RNase T1 (Fig. 7A). For RNase 4, NASE revealed a 33.3% and BPF 29.6% sequence coverage and most cleavage sites were the expected UΙG and UΙA sites (Fig. 7A) Only 12 detected cleavage sites out of 90 were off-target cleavages with UΙC, CΙG and AΙA sites dominating (Fig. 7B).

For colicin E5, NASE found a sequence coverage of 43.6% and BPF of 29.49%. As shown in Fig. 7B, over 100 off-target sites are detectable for colicin E5. Next to GΙU, high cleavage rates are observed for UΙU and AΙU sites (Supplementary Fig. S22). While individual RNases provide incomplete and partially redundant sequence coverage, their combination significantly increases the fraction of uniquely identifiable sequence regions in mRNA to 79.8% according to NASE (Fig. 7C) and 67.9% according to BPF (Supplementary Fig. S23). Interactive coverage maps containing information for each nucleotide position are provided as HTML files (Supplementary data). A detailed list of all detected fragments from NASE is given in Supplementary Tables S6 and S7, while all detected fragments from BioPharma Finder are listed in Supplementary Table S8.

## Discussion

Our results establish a nanogram-sensitive workflow for oligonucleotide MS that integrates controlled RNA fragmentation with ion-pair–free nanoflow HILIC separation. By addressing both fragmentation and chromatographic sensitivity as interdependent parameters, this approach extends the applicability of RNA MS to low-input and native RNA samples and enables its use as an orthogonal tool to sequencing-based technologies.

Compared to conventional analytical- and microflow-based approaches, the nanoflow HILIC setup reduces RNA input requirements by approximately one to two orders of magnitude (Supplementary Fig. S25). This improvement enables analysis of tens of nanograms of RNA, which was previously not feasible in most standard oligonucleotide LC-MS workflows [18]. Notably, this performance is comparable to existing nanoflow methods that rely on IP reagents [23, 39], while avoiding their inherent limitations in LC-MS compatibility and robustness.

This gain in sensitivity comes at the cost of reduced system robustness, including increased susceptibility to leaks and spray instability. Therefore, the nanoflow setup is particularly suited for applications where sensitivity is the primary constraint, rather than for routine high-throughput analyses.

The choice of RNase emerged as a critical determinant of data quality and sequence coverage. While RNase T1 provides robust and predictable cleavage, it inherently restricts sequence accessibility due to formation of non-unique fragments. In contrast, RNase 4 and colicin E5 expand accessible sequence space but introduce variability through context-dependent and off-target cleavage.

These findings demonstrate that RNA fragmentation is not a trivial preprocessing step but a central parameter that must be actively controlled and adapted to the analytical objective. In particular, the balance between specificity and coverage represents a key trade-off in oligonucleotide MS.

Regarding the substrate specificity, we find that RNase T1 remains to be the most reliable RNase in terms of substrate specificity. Colicin E5, originally described as a motif-specific nuclease targeting YGUN sequences [34], exhibited substantially broader cleavage behavior in our experiments, including frequent off-target activity. RNase 4 also showed deviations from its reported specificity, albeit to a lesser extent. These observations highlight that RNase behavior is highly dependent on sequence context and experimental conditions and cannot be fully predicted from literature reports alone. From a practical perspective, RNase T1 remains the most reliable choice for defined RNA substrates, whereas RNase 4 and colicin E5 can be advantageous for expanding sequence coverage in purified systems. However, their use in complex RNA mixtures is likely limited by increased ambiguity in fragment assignment.

In general, and especially for colicin E5, we recommend the use of alkaline phosphatase [40] for generation of uniform 3′-hydroxylated oligonucleotides. In this benchmarking study, we intentionally omitted a dephosphorylation step to allow for a comprehensive characterization of the raw enzymatic output. While adding a phosphatase is a common strategy to simplify MS spectra by merging cyclic, linear, and non-phosphorylated species, our approach provides critical insights into the 3'-end chemistry produced by each RNase. We observed that the distribution between 2',3'-cyclic and 3'-linear phosphates varies significantly between enzymes, which is of high practical importance for downstream protocol design. For instance, while calf intestinal phosphatase is highly efficient for linear 3'-phosphates, T4 polynucleotide kinase is required for the effective removal of cyclic phosphates [57]. By establishing the specific 3'-termini, we enable researchers to select the most appropriate phosphatase for their specific RNA substrates, ultimately maximizing signal intensity and sequence coverage in future applications.

The presented data demonstrate compatibility with modification-containing RNAs, including both synthetic pseudouridine-containing substrates and native tRNA. However, systematic assessment across diverse modification classes remains an open challenge.

While the presented workflow addresses key limitations in sensitivity and fragmentation control, several analytical challenges remain. Regarding the size limitations of the presented workflow, the longest fragment successfully identified in this study was a 41-mer. While our workflow provides high sensitivity, the analysis of longer oligonucleotides (typically >40 nucleotides) poses several technical challenges. First, chromatographic separation of longer oligonucleotides remains limited by strong retention on commonly used HILIC materials [42, 58]. Second, ionization efficiency in the electrospray source decreases with increasing chain length and water content. On top of that, larger oligonucleotides are more prone to cation adduct formation (e.g. Na⁺, K⁺), which complicates the mass spectra by splitting the signal into multiple peaks and increasing the complexity of data deconvolution. Consequently, the technical ceiling for robust sequence mapping currently remains in the range of short to medium length oligonucleotides due to these cumulative physico-chemical constraints. In addition, differentiation of isobaric and positional RNA modifications remains dependent on fragmentation behavior [20] and is not yet universally resolved. Furthermore, quantitative analysis of oligonucleotides is currently not routinely achievable in this workflow and will require the development of appropriate normalization or labeling strategies.

Future developments will need to address quantitative analysis, improved control of enzymatic fragmentation, and the resolution of isobaric and positional RNA modifications. Integration of these aspects will be essential for establishing oligonucleotide MS as a routine analytical platform. Together, these results establish RNase fragmentation control and chromatographic sensitivity as jointly defining parameters for oligonucleotide MS workflows.

## Supplementary Material

gkag691_Supplemental_Files

## Data Availability

The oligonucleotide MS data underlying this article have been deposited to the ProteomeXchange Consortium via the PRIDE [59] partner repository (https://www.ebi.ac.uk/pride/) with the dataset identifier PXD070755.
