# Supplementary material for: A nanogram-sensitive workflow for oligonucleotide mass spectrometry using ion-pair-free nanoflow HILIC and RNase benchmarking
Source: Nucleic Acids Res. 2026 Jul 14;54(13):gkag691. doi: 10.1093/nar/gkag691 (PMC13366003; doi:10.1093/nar/gkag691)
Supplement: gkag691_Supplemental_Files [file gkag691_supplemental_files.zip › Qi et al SI.pdf]

Supplementary Information

A Nanogram-Sensitive Workflow for Oligonucleotide Mass Spectrometry Using Ion-Pair–Free Nanoflow HILIC and RNase Benchmarking

Yuyang Qi[§,1], Chengkang Li[§,*,1,2,3], Nur Yesiltac-Tosun[§,1], Jannick Schicktanz[1], Leona Rusling[1,4], Steffen Kaiser[4], Samuel Wein[5,6], and Stefanie Kaiser*[,1]

Affiliations:

[1] Department of Pharmaceutical Chemistry, Goethe University Frankfurt, Frankfurt, 60438, Germany;

[2] Pediatric Cancer Metabolism Laboratory, Children's Research Center, University of Zurich, 8008 Zurich, Switzerland;

[3] Division of Oncology, University Children's Hospital Zurich and Children's Research Center, University of Zurich, 8008 Zurich, Switzerland;

[4] Mass Spectrometry Service Unit, Goethe-University Frankfurt, Max-von-Laue-Str. 9, 60438 Frankfurt/M., Germany

[5] Applied Bioinformatics, Dept. of Computer Science, University of Tübingen, Tübingen, 72074, Germany;

[6] OpenMS Inc., Erie, Pennsylvania 16502, United States

§ contributed equally to the manuscript

Corresponding authors email address: chengkang.li@kispi.uzh.ch and stefanie.kaiser@pharmchem.uni-frankfurt.de

Table S1 Overview of RNases commonly used for oligonucleotide MS.

RNase	Cleavage motif		Specificity class	Modification tolerance	Key limitations in MS analysis
**RNase T1**	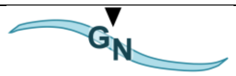	[4] [5] [6] [7]	High (single nucleotide)	Accepts inosine as substrate Limited (blocked by 2′-O-methylation) [9]	Short fragments, reduced sequence coverage
**RNase U2**	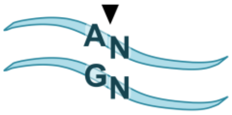	[3] [7] [11]	Moderate	Variable (tolerates some modifications, e.g. Ψ, m2,2G) [7]	Broader specificity increases search space
**RNase T2**	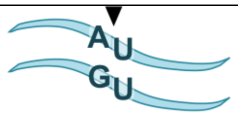	[14] [15] [16]	Low	Limited (affected by 2′-O-methylation)	Unspecific cleavage, complex fragment mixtures
**RNase A**	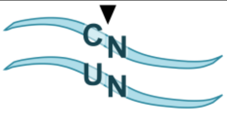	[23] [24] [25] [26]	Moderate	Accepts e.g. D, m5C, m5U and Ψ; blocked by 2′-O-methylation [9] [24]	Short fragments, incomplete coverage
**RNase 4**	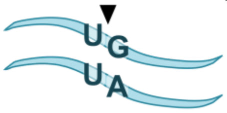	[6]	Moderate	Accepts $m^1Ψ$ and D; blocked by 2′-O-methylation) [6]	Sequence bias, variable efficiency
**MC1**	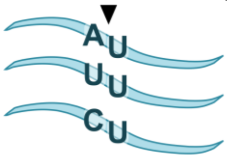	[18]	High (motif-based)	Limited (many modifications reduce cleavage efficiency) [18], [19], [6]	Strong sequence constraints, incomplete coverage
**Cusavitin**	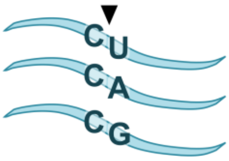	[18]	High (motif-based)	Blocked by several base and ribose modifications [20]	Restricted cleavage sites
**MazF**	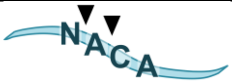	[5] [29] [30] [31]	High (motif-specific)	Blocked by m^6A [32]	Very narrow sequence window
**Colicin E5**	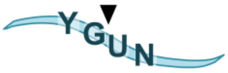	[5] [33] [34]	High (motif-specific)	Limited (affected by ribose methylation) [35]	Narrow specificity, potential over-fragmentation

Table S2 Reported flow rates and injection amounts in IP-RP-LC-MS

Flow regime	Sample type	Typical input	Limitation
Analytical	Synthetic	5-20 pmol [36-40]	Low sensitivity
	hydrolysate	0.7-20 µg [5,41,42]	
Microflow / Cap flow	Synthetic	1.2-7.5 pmol [43,44]	Moderate sensitivity
	hydrolysate	3-8 µg [12,45]	
Nanoflow	Synthetic	0.3-50 fmol [46,47]	Robustness / variability

	hydrolysate	5 ng – 10 µg [46-50]	

Table S3 RNA and DNA used in this manuscript

name	sequence (5‘ to 3‘)	supplier
5 mer	AUUUG	IBA
8 mer	UUUCCCCG	IBA
9 mer	GUUUCCGUA	Sigma
10 mer	AAAUCCAUUG	IBA
13 mer	GGCGGAAACACCA	Sigma
20 mer	UGAGGCAGGAGGUUGAAUAG	Dharmacon
30 mer	AAGCUGAGGCAGGAGGUUGAAUAGCAUGCA	Dharmacon
40 mer	GUA GUC GUG GCC GAG UGG UUA AGG CGA UGG ACU UGA AAU C	IBA
DNA T10	TTTTTTTTTT	Sigma
DNA T15	TTTTTTTTTTTTTTT	Sigma
DNA T20	TTTTTTTTTTTTTTTTTTTT	Sigma
DNA T30	TTTTTTTTTTTTTTTTTTTTTTTTTTTTTT	Sigma
DNA T50	TTTTTTTTTTTTTTTTTTTTTTTTTTTTTTTTTTTTTTTTTTTTTTTTTT	Sigma
77 mer $tRNA^{Ile}$	GGCUUGUAGCUCAGGUGGUUAGAGCGCACCCCUGAUAAGGGUGAGGUCGGUGGUUCAAGUCCACUCAGGCCCACCA	IVT (in house)

Table S4 defined parameter for RNase T1, RNase 4 and colicin E5 for the test of different reaction conditions

Nuclease	Enzyme to substrate ratio	Temperature [°C]	Incubation time [min]	buffer
RNase T1	10 U/µg tRNA	37	30	90 mM NaCl + 25 mM Tris HCl pH 7.5
RNase 4	15 U/µg tRNA	37	60	NEBuffer^{TM} r1.1*
Colicin E5	4 mol Colicin E5 to 1 mol substrate	37	30	90 mM NaCl + 25 mM Tris HCl pH 7.5

*contains 10 mM Bis-Tris-Propane-HCl, 10 mM $MgCl_2$, 100 µg/ml Recombinant Albumin, pH 7.0@25°C

Table S5. Calculated limit of detection (LoD) and limited of quantification (LoD).*

	dT10	dT15	dT20	dT30	dT50
LoD (pmol)	0.21	0.39	0.23	0.40	0.27
LoQ (pmol)	0.63	1.19	0.70	1.22	0.81
LoD (fmol/nt)	20.78	26.21	11.54	13.46	5.36
LoQ (fmol/nt)	62.98	79.43	34.98	40.80	16.24
LoD (ng/nt)	0.06	0.12	0.07	0.12	0.08
LoQ (ng/nt)	0.19	0.36	0.21	0.37	0.25

*Calibration curves were prepared using mixtures of DNA oligonucleotides, including dT10, dT15, dT20, dT30, and dT50, in different injection amounts, i.e. 0.2, 0.4, 1, 2, and 4 pmol.

Table S6. Detected cleavage fragments from FLuc mRNA, calculated with NASE. Fragments with * are unspecific uncleavage products.

RNase T1	colicin E5	RNase 4
AAAAAGp	UAACAACCGCGAAAAAGc	AAAACGGAUUc
AAAAAGUUGCGCGGAGp	UACCAGAGc	AAAGAAAGGCCCGGCGCCAUUCUc
AAAAAUCAGp	UACCCCCGc	AACAACCGCGAAAAAGUUc
AAAACGp	UACCGAAAGGc	AAGAAGAAAUc
AAAACUCGp	UAUGCAGc	AAUACGACUCACUAUc
AAAACUCUCUUCAAUUCUUUAUGp	UCACAUCUCAUCUACCUCCCGGc	AAUCCAGAAAAUUc
AAAAGp	UCGCCAGc	ACAAAAUUCAAAGUc
AAAAUUAUUAUCAUGp	UCGGGGAAGCGGc	ACAAGCGAUc
AAAGp	UCUUCCCGACGAUGACGCCGGc	ACACCCGAGGGGGAUc
AAAUAUAAGp	UGAUAAUAGGCUGGAGCCUCGGc	ACACGAAAUUc
AACAAUUGp	UGGACAUCACGc	ACACUCGGAUc
AACACUUCUUCAUAGp	UGGACGAAGc	ACCAACCCUc
AACGp	UGGAUUACGc	ACCCCCGUc
AACUCCUCUGp	UGGCAGGc	ACCGAAAGGUCUUc
AACUUCCCGp	*AAAAAAAAUc	ACCGGAUACCUc
AAGGGCGGAAAGAUCGp	*AAAACGCUc	ACCGGGAAAACGCUc
AAUAAAGp	*AAAUACGAc	ACCUCCCGGUUUUc
AAUACAAAUCACAGp	*AAAUACGAUc	ACGACAAGGAUc
AAUACUUCGp	*AACCCUAUc	ACGAUCCCUUCAGGAUUc
AAUAUGp	*AAGAAAAAUCc	ACGCGGAAUc
AAUUAUGp	*ACAAAAAUc	AGGCUGGAGCCUCGGUc
ACAAAACAAUUGp	*ACGAAAUc	GAACUCCUCUc
ACAAAUACGp	*ACUCUGAUc	GAAGAGGAGCUc
ACAAGp	*AGAAUCGc	GAAGCGAAGGUUc
ACAUCACGp	*AUCAAAAUc	GACAAAACAAUUc
ACAUUUAUAAUGp	*CAAAAAAAAc	GACGCCGGUc
ACCAACGp	*CAAAAAAc	GACGGAAAAAGAGAUCGUc
ACCUAUGp	*CAAAAAAUc	GAGACUACAUCAGCUc
ACUACAUCAGp	*CAAAAAAUUc	GCAAAAAAAAUUc
AUAAACCGp	*CAAAACGCUc	GCAAAAAAUUUUc
AUAACGp	*CACCCGc	GCACCCGUc
AUAAUAGp	*CACCUCUc	GCCAGAGAUCCUc
AUAAUGp	*CACUAUAGGGc	GCGUCAGAUUCUCGCAUc
AUACCAGp	*CACUGAUAAc	GGAACCGCUc
AUACGp	*CAGCCUACCGc	GGAAGACGCCAAAAACAUc
AUACUGp	*CAUAGAACUGc	GGACAUCACGUc
AUAGp	*CAUCAGGAGc	GGAGAGCAACUc
AUAUGp	*CAUCAGGAGUc	GGAGCCUCGGUGGCCUc

AUAUUGp	*CAUCGAGc	GGCAGAAGCUc
AUAUUUGp	*CGAAAAAGc	GGCCCCCGCUc
AUCCCUUCAGp	*CGGAAUACUc	GGCCCUUCCGCAUc
AUCCUAUUUUUGp	*CUCAACAGc	GGGCAUUUCGCAGCCUc
AUCCUCAUAAAGp	*CUCAUCUc	GGGCGCGUUc
AUCUACUGp	*GAAAGAAGc	GGGCUCACUc
AUCUUCACCUAGp	*GUCAAAAAUAc	GGGCUCACUGAGACUc
AUUACAAAAUUCAAAGp	*UAAAGc	GGUUCCUGGAACAAUUc
AUUACACCCGp	*UAAGAAGAAAc	GUCAGAGGACCUc
AUUACCAGp	*UAAUGAACGc	GUUCCAUUCCAUCACGGUUUUGGAAUc
AUUAUGp	*UACAAAAc	*CAUUCCGGAUc
AUUCUAAAACGp	*UACAAAAUc	*CUUCCAGGGAUc
AUUCUCGp	*UACACGAAAc	*GGGCGGCc
AUUUAUCUAAUUUACACGp	*UACACGAAAUc	*ACCGGAAAACUc
AUUUCAGp	*UACACGc	*CGACGCAAGc
AUUUCGp	*UACAGAUGc	*GGGCGCGc
AUUUUAAGp	*UACCAACCCUAc	*UUUUGGCAAc
AUUUUGp	*UACCAAUAAc	
CAAAAAAAAUUACCAAUAAUCCAGp	*UACCAGGGAc	
CAAAAAAUUUUGp	*UACCAGGGAUc	
CAAAACGp	*UACCUAAGGGc	
CAACGp	*UACGAUCCCUc	
CAACUGp	*UACGCCCUGGc	
CAAUCAAAUCAUUCCGp	*UACUGCGAUc	
CACAUAUCGp	*UACUUCGAAAc	
CACCCGp	*UAUAAUGAACGc	
CACCCGUACCCCCGp	*UAUAGAUc	
CACCUCUUUCGp	*UAUCAUGGAc	
CACUCUGp	*UAUCAUGGAUc	
CACUGp	*UAUCGGAGc	
CAGCAGp	*UAUCUAAUc	
CAGUGGp	*UAUGCCGGc	
CAUAAGp	*UAUGGGCAUc	
CAUAGp	*UCAAAGc	
CAUCGp	*UCAAAUCAc	
CAUGp	*UCAAAUCAUc	
CCAAAAACAUAAAGp	*UCAAUUCUc	
CCAAAAGp	*UCACAGAAUCGc	
CCAAGp	*UCACAUCUCAc	
CCACCAUGp	*UCACAUCUCc	
CCAUUCUAUCCGp	*UCACGUUAc	
CCCCCCUGp	*UCAUGGAUc	
CCCCCGp	*UCCAUCACGc	
CCCCUUGp	*UCCAUCACGGc	
CCCUUCCGCAUAGp	*UCCGGc	
CCCUUCCGp	*UCCUGCACCCGc	
CCUACCGp	*UCCUGGAACAAc	
CCUCGp	*UCGAAAGAAGc	
CCUUGp	*UCGAGc	
CGCCAUUCUAUCCGp	*UCGAUAUc	
CUACAGp	*UCGGAGc	
CUACAUUCUGp	*UCUAAAACGGAc	
CUAUGp	*UCUCGc	
CUAUUCUGp	*UCUCGCAc	
CUCAACAGp	*UCUCGCAUGc	
CUCACUGp	*UCUGAUc	
CUUACUGp	*UCUUCAUAGc	
CUUCCAUCUUCCAGp	*UGAACGc	
CUUCUGp	*UGAACUc	
CUUCUUGp	*UGAACUUCCc	

CUUUUACAGp	*UGAAGCGAAGc	
GAUUAGCAGAGCGAGGp	*UGAAGCGAAGGc	
GAUUAGCAGAGCGAGGUAUGp	*UGAAUAAAGc	
UAAAAAGp	*UGAAUACGAc	
UAAACAAUCCGp	*UGAAUACGAUc	
UAAAGp	*UGACAAAACAAc	
UAACAACCGp	*UGACAAAUc	
UAACUAUCGp	*UGACAAGGAc	
UACACGp	*UGACCGCUc	
UACCAACCCUAUUUUCAUUCUUCGp	*UGAUAc	
UACCCCCGp	*UGAUCGc	
UACGp	*UGCAAAAAAAAc	
UAUGp	*UGCAAAACGCUc	
UCACAUCUCAUCUACCUCCCGp	*UGCAGc	
UCCUUUGp	*UGCCCCUc	
UCUUAAUGp	*UGCUAGc	
UCUUACCGp	*UGCUCAACAGc	
UCUUCCCGp	*UGGAAc	
UCUUUAAUUAAAUACAAAGp	*UGGAAUGc	
UCUUUGp	*UGGAGc	
UUAAUCAGp	*UGGAUUCUAAAc	
UUACAACACCCCAACAUCUUCGp	*UGGCAAUCc	
UUACCUAAGp	*UGGCCCUc	
UUAUGp	*UGGCCUAGCUc	
UUAUUGp	*UGGGCAUc	
UUAUUUAUCGp	*UGGGCGc	
UUCCAUAGp	*UGGGCGCGc	
UUCCAUUCCAUCACGp	*UGGGCGGCc	
UUCCAUUUUUUGp		
UUCCGACCCUGCCGp		
UUCCUGp		
UUUACUACACUCGp		
UUUCCAAAAAGp		
UUUCCCCUGGAAGCUCCCUCGp		
UUUCCCGp		
UUUUAAUGp		
UUUUGp		
UUUUUACGp		

Expression and Purification of Colicin E5

Map of plasmid encoding CE5 and IMM5:
HMLAKNKGKIPGLKIDQKIRGQMPERGWTEDDIKNTVSNGATGTSFDKRSPKKTPPD
YLGRNDPATVYGSPGKYVVVNDRTGEVTQISDKTDPGWVDDSRIQWGNKNDQ*

>His-TEV-CE5-Spacer-T7 promoter-T7 gene 10 RBS-Im5
ATGCATCACCATCACCATCACgaaaacctgtattttcagagcCTGGCTAAAAACAAAGGTAAAATCCCGG
GTCTGAAATCGACCAGAAAATCCGTGGTCAGATGCCGGAACGTGGTTGGACCGAAGACGACATCAAAAAC
ACCGTTTCTAACGGTGCTACCGGTACCTCTTTCGACAAACGTTCTCCGAAAAAAACCCCGCCGGACTACC
TGGGTCGTAACGACCCGGCTACCGTTTACGGTTCTCCGGGTAAATACGTTGTTGTTAACGACCGTACCGG
TGAAGTTACCCAGATCTCTGACAAAACCGACCCGGGTTGGGTTGACGACTCTCGTATCCAGTGGGGTAAC
AAAAACGACCAGTAAGTAAATTCTCGGCGTGCTTGGTGTCTCGTATTTCTCCTGGAGTAGAAGCATTAAT
ACGACTCACTATAGaataattttgtttaactttaaaaaaaaaaaagaaggagaataatctATGACCAACA
AACTGTTCGAACACACCGTTCTGTACGACTCTGGTGACGCTTTCTTCGAACTGAAAGGTAACGCTTCTAT
GAAACTGTCTCCGAAAGCTGCTATCGAAGTTTGCAACGAAGCTGCTAAAAAAGGTCTGTGGATCCTGGGT
ATCGACGGTGGTCACTGGCTGAACCCGGGTTTCCGTATCGACTCTTCTGCTTCTTGGACCTACGACATGC
CGGAAGAATACAAATCTAAAATCCCGGAAAACAACCGTCTGGCTATCGAAAACATCAAAGACGACATCGA
AAACGGTTACACCGCTTTCATCATCACCCTGAAAATGTAA

Due to the ribonuclease activity of Colicin E5 and its toxicity to *E. coli*, it was coexpressed

with Imm5, which neutralizes Colicin E5 by direct binding. The sequences of the open reading frames (ORFs) for Colicin E5 and Imm5 are provided in the 5′–3′ direction in both nucleotide (RNA) and amino acid formats.

The plasmid was transformed into chemically competent *E. coli* BL21(DE3) Rosetta cells. Competent cells (50 µL) were mixed with 1 µL of plasmid DNA (30–100 ng) and incubated on ice for 20 minutes. Transformation was achieved by heat-shocking the cells at 42°C for 50 seconds, followed by immediate cooling on ice for 2 minutes. Subsequently, 100 µL of antibiotic-free 2 LB medium was added, and the cells were incubated at 37°C for 1 hour. Approximately 30 µL of the culture was plated on LB-agar containing 50 µg/mL kanamycin and incubated overnight at 37°C.

A single colony was used to inoculate 100 mL of TB medium supplemented with chloramphenicol (0.034 mg/mL) and kanamycin (0.1 mg/mL). The pre-culture was grown overnight at 37°C and 200 rpm. For large-scale expression, 12 mL of pre-culture was added per 1 L of TB medium. Cultures were grown at 37°C, 200 rpm until OD600 reached 1.5. At this point, the temperature was reduced to 18°C and shaking speed to 130 rpm. When OD600 reached 3.5, protein expression was induced with 500 µL/L of 1 M IPTG. Cultures were incubated overnight under these conditions.

Cells were harvested by centrifugation at 6000 rpm and 4°C for 15 minutes using a JLA 8.1000 rotor. From 8 L of culture, approximately 16 g of cell pellet per liter was obtained. Half of the harvested biomass was resuspended in 160 mL of lysis buffer. Cells were lysed by sonication (2 × 9 min cycles), and nucleic acids were precipitated by adding 4.4 mL of polyethyleneimine (PEI), followed by centrifugation at 23,000 × g for 40 minutes. The resulting clear supernatant was used for protein purification.

Purification was performed using a 5 mL Immobilized Metal Affinity Chromatography (IMAC) column on an ÄKTAprime liquid chromatography system. The column was equilibrated with lysis buffer, and the filtered supernatant (0.45 µm) was loaded at 3 mL/min. After washing with 10 mM imidazole buffer to reach baseline, the column was sequentially washed with 50 mL each of Refolding Buffer 1 and Refolding Buffer 2. Colicin E5 was eluted using a linear gradient from 0 to 300 mM imidazole over 60 mL. Samples from purification steps were analysed by SDS-PAGE.

Further purification was performed via size-exclusion chromatography (SEC) using a Superdex™ 75 column. Fractions containing Colicin E5 were pooled, concentrated, and purified to homogeneity. The final protein concentration was 3.5 mg/mL, yielding approximately 20 mg of purified Colicin E5.

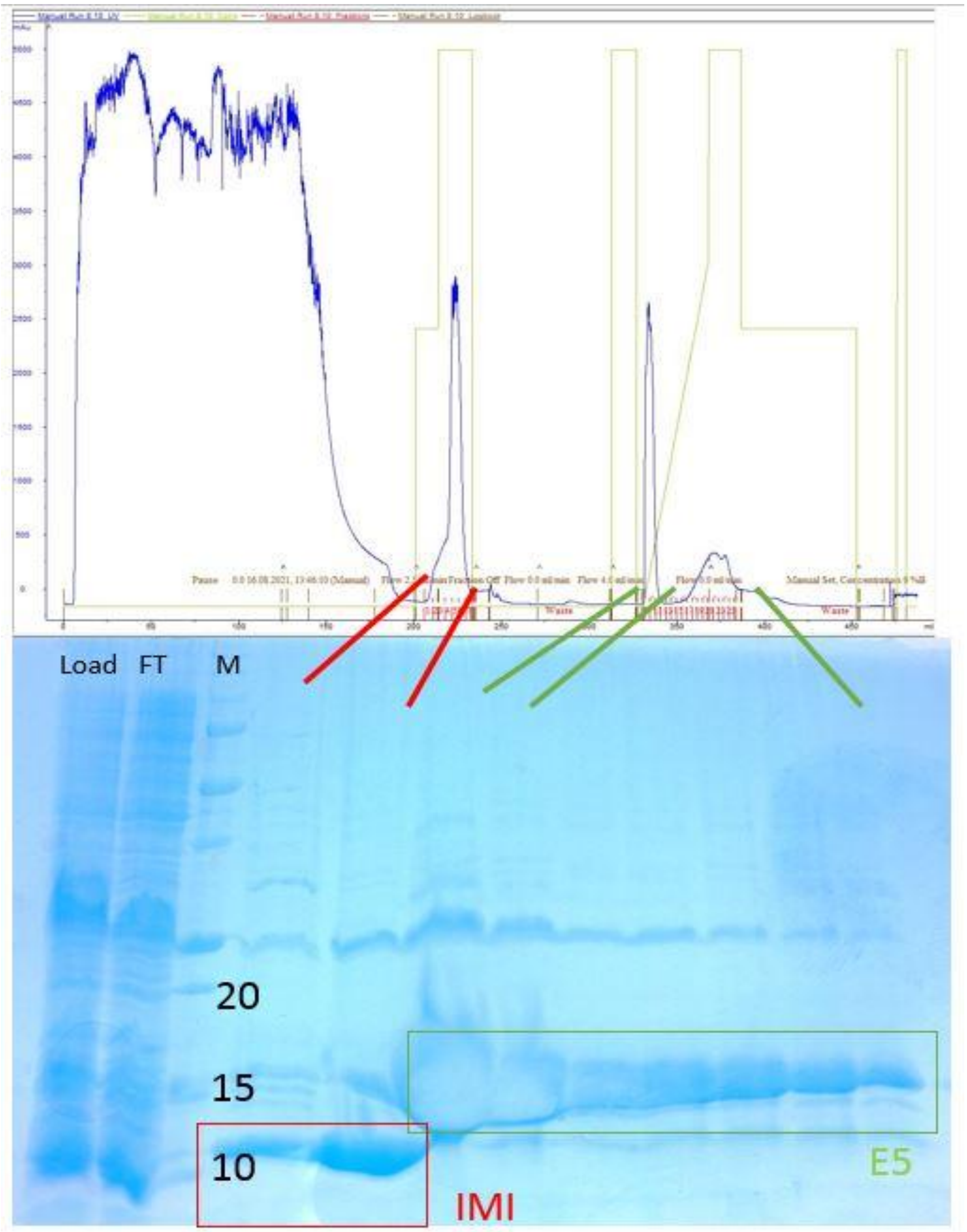

Figure S1: Purification was performed using a 5 mL Immobilized Metal Affinity Chromatography (IMAC) column on an ÄKTAprime liquid chromatography system. The column was equilibrated with lysis buffer, and the filtered supernatant (0.45 µm) was loaded at 3 mL/min. After washing with 10 mM imidazole buffer to reach baseline, the column was sequentially washed with 50 mL each of Refolding Buffer 1 and Refolding Buffer 2. Colicin E5 was eluted using a linear gradient from 0 to 300 mM imidazole over 60 mL. Samples from purification steps were analysed by SDS-PAGE. Red Box labelled IMI: Imm5, Green box labelled E5: colicin E5.

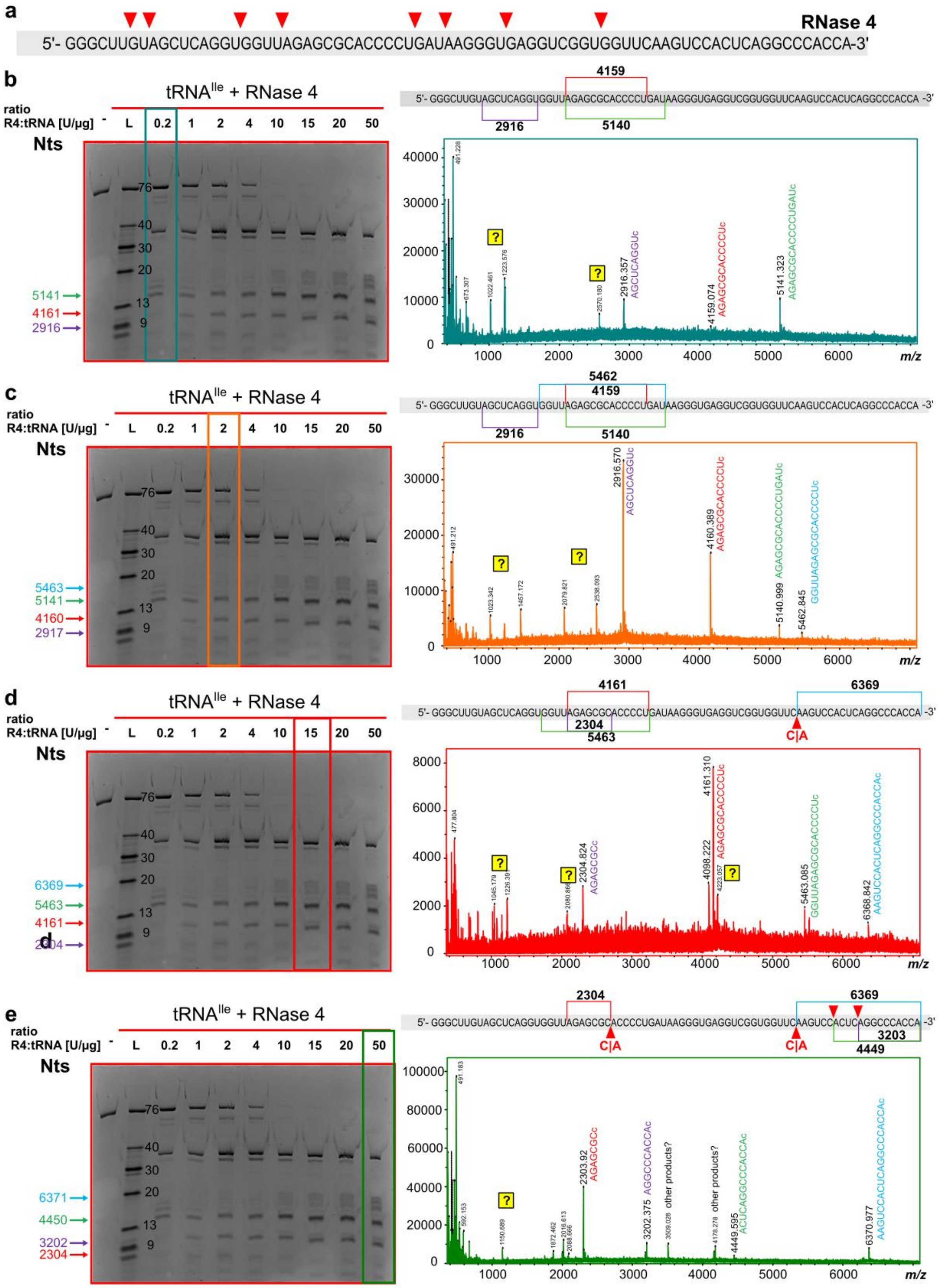

Figure S2: Substrate specificity of RNase 4 at different enzyme to substrate ratios. a) sequence of an unmodified tRNA (E. c. tRNA-Ile-GAU) and the expected cleavage sites of RNase 4 hydrolysis. b-e) polyacrylamide gel of RNase 4 digested E. c. tRNA-Ile-GAU with different enzyme to substrate ratios and the corresponding MALDI-MS spectra. Assigned fragments and cleavage sites are added to the MS spectrum and in the sequence above. c = 3' cyclic phosphate.

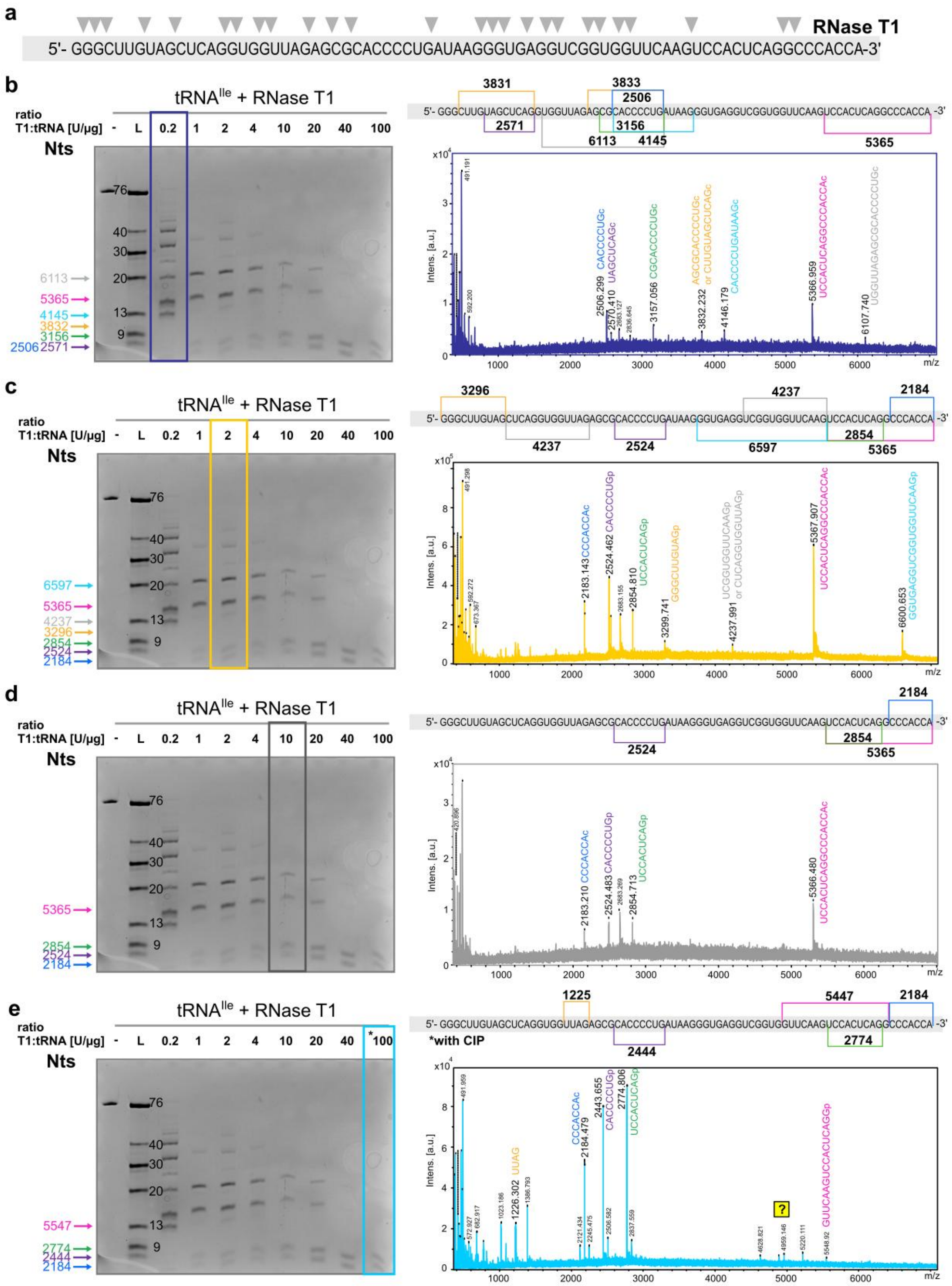

Figure S3: Substrate specificity of RNase T1 at different enzyme to substrate ratios. a) sequence of an unmodified tRNA (E. c. tRNA-Ile-GAU) and the expected cleavage sites of RNase T1. b-e) polyacrylamide gel of RNase T1 digested E. c. tRNA-Ile-GAU with different enzyme to substrate ratios and the corresponding MALDI-MS spectra. Assigned fragments and cleavage sites are added to the MS spectrum and in the sequence above. c = 3’ cyclic phosphate and p = 3’ linear phosphate.

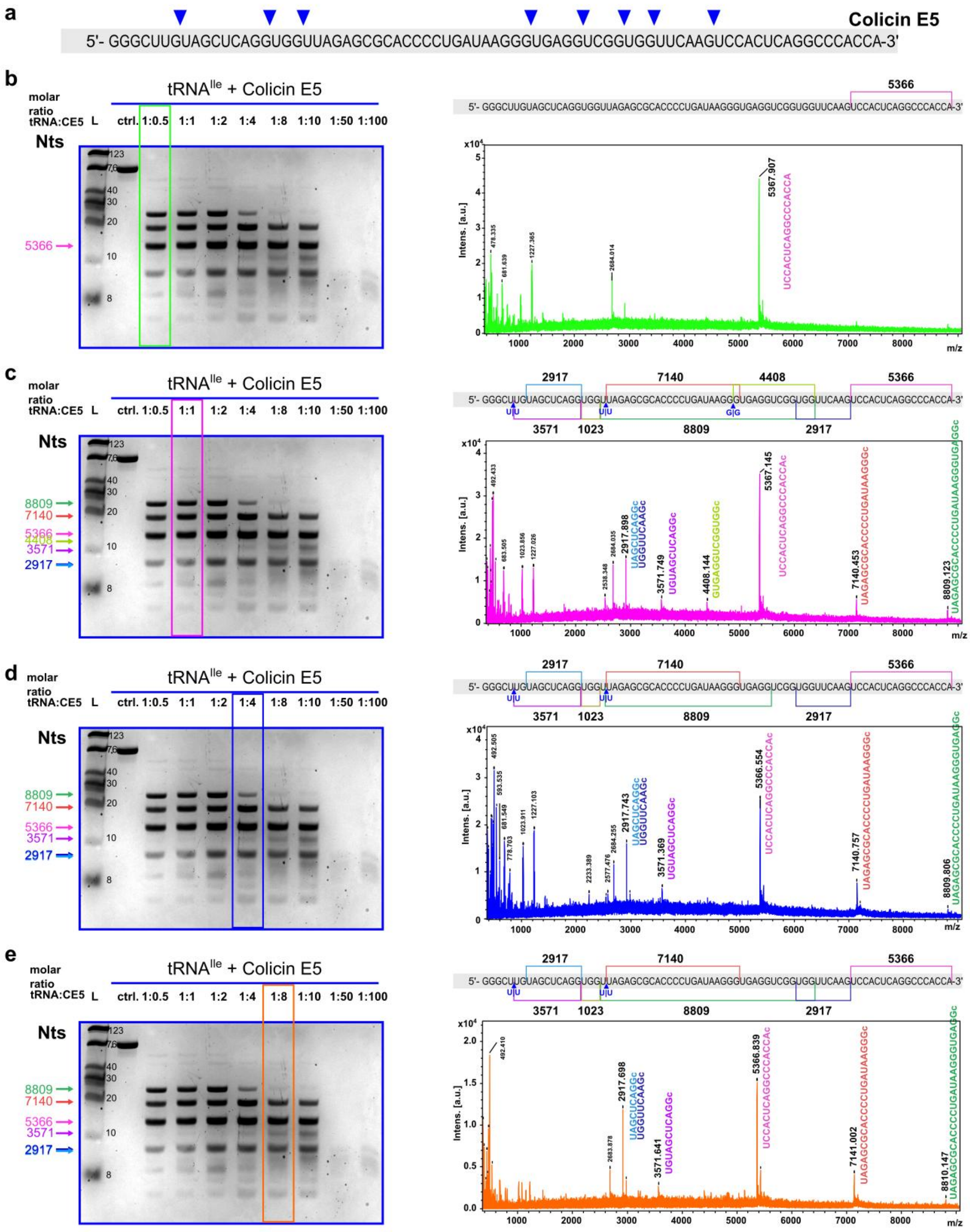

Figure S4: Substrate specificity of colicin E5 at different enzyme to substrate ratios. a) sequence of an unmodified tRNA (E. c. tRNA-Ile-GAU) and the expected cleavage sites of colicin E5. b-e) polyacrylamide gel of colicin E5 digested E. c. tRNA-Ile-GAU with different enzyme to substrate ratios and the corresponding MALDI-MS spectra. Assigned fragments and cleavage sites are added to the MS spectrum and in the sequence above. c = 3' cyclic phosphate.

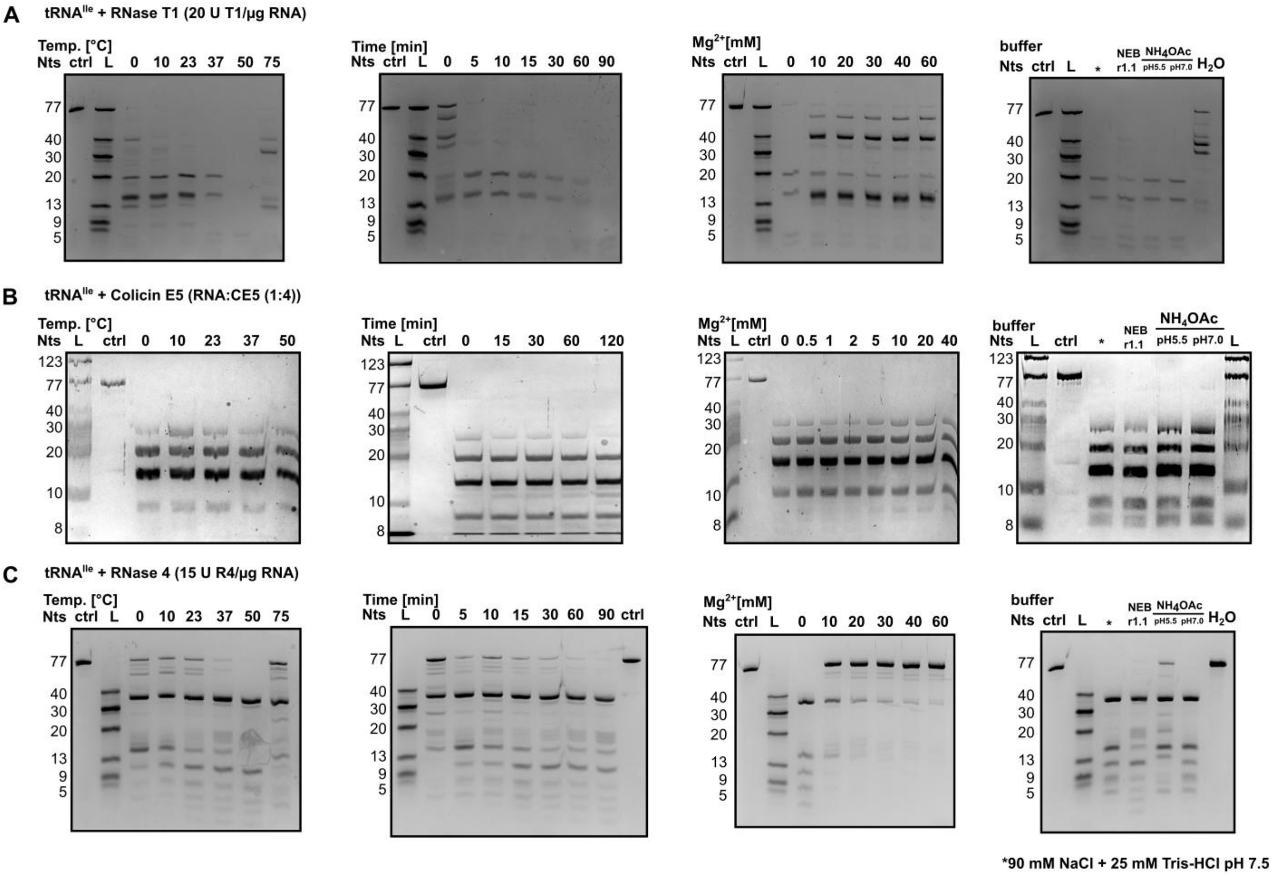

Figure S5: Variability of digestion pattern with different reaction conditions. Polyacrylamide gel of E. c. tRNA-Ile-GAU treated with different temperature, incubation time, buffer composition and pH with **A**) RNase T1 **B**) colicin E5 and **C**) RNase 4.

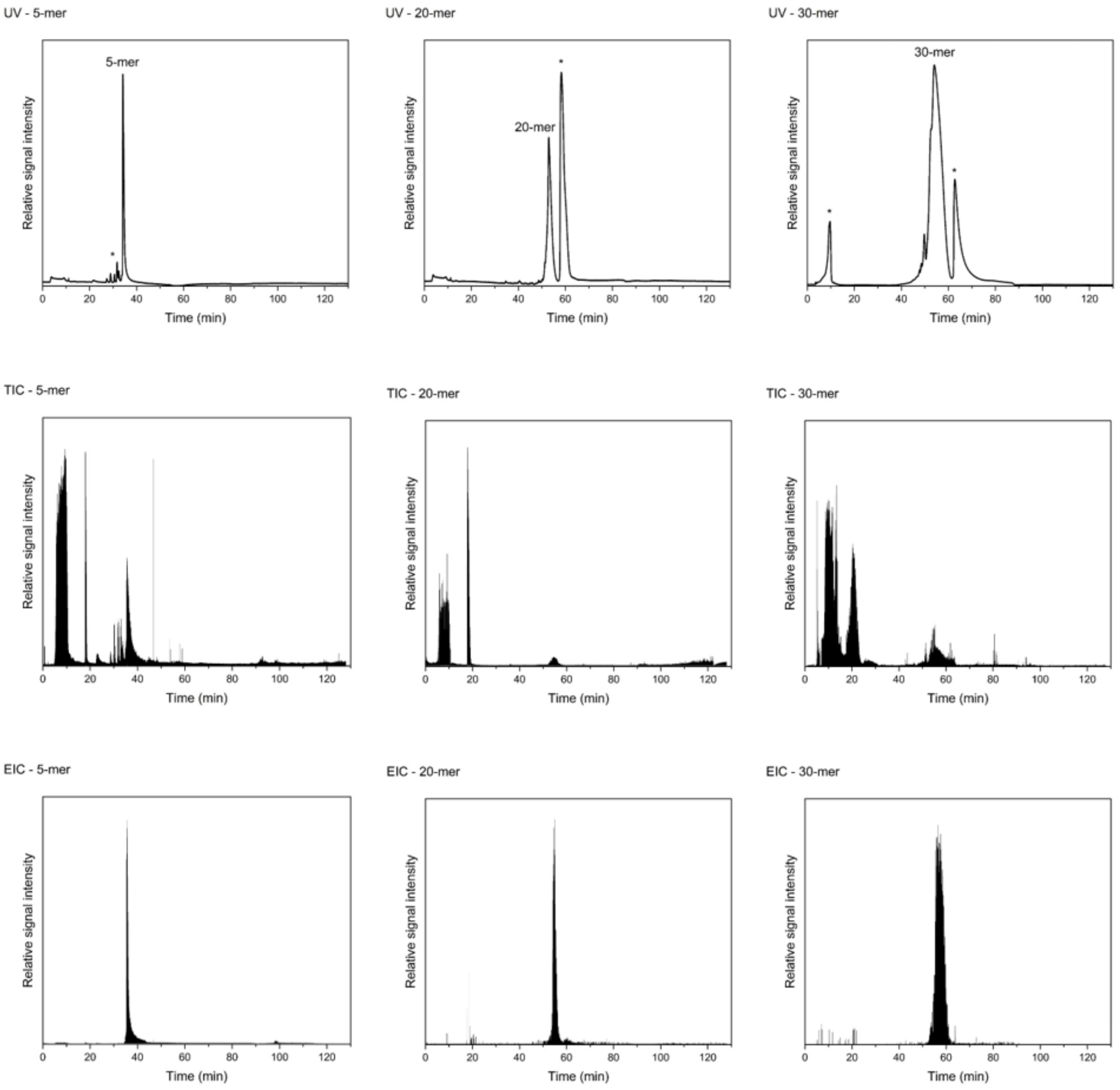

Figure S6 UV chromatogram, Total ion chromatograms (TICs), and extracted ion chromatograms (EICs) of a 5-mer, a 20-mer, and a 30-mer (synthetic ribonucleotides) analyzed under positive ion mode (POS) via direct injection (DI). Impurities were present in these samples as marked by the asterisk (*) signs in the UV chromatograms.

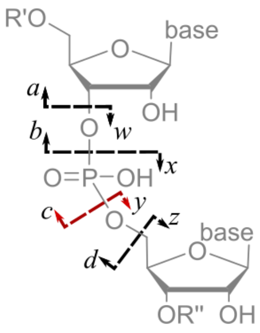

Figure S7 Nomenclature of MS/MS fragments in oligonucleotide mass spectra. (51)

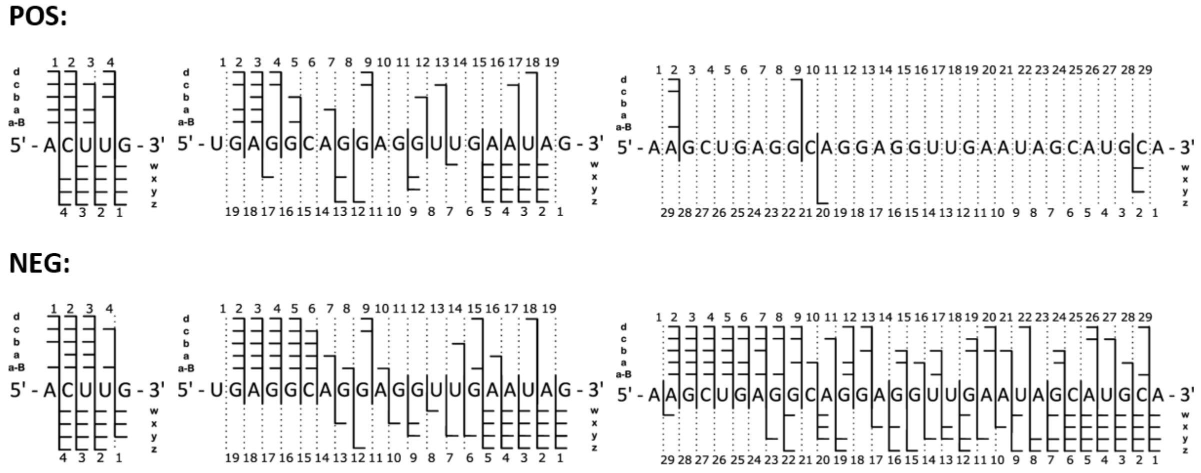

Figure S8 Sequence coverage of a 5-mer, 20-mer and 30-mer after ionization in positive ion mode (POS) and negative ion mode (NEG). Coverage was determined using NASE (15).

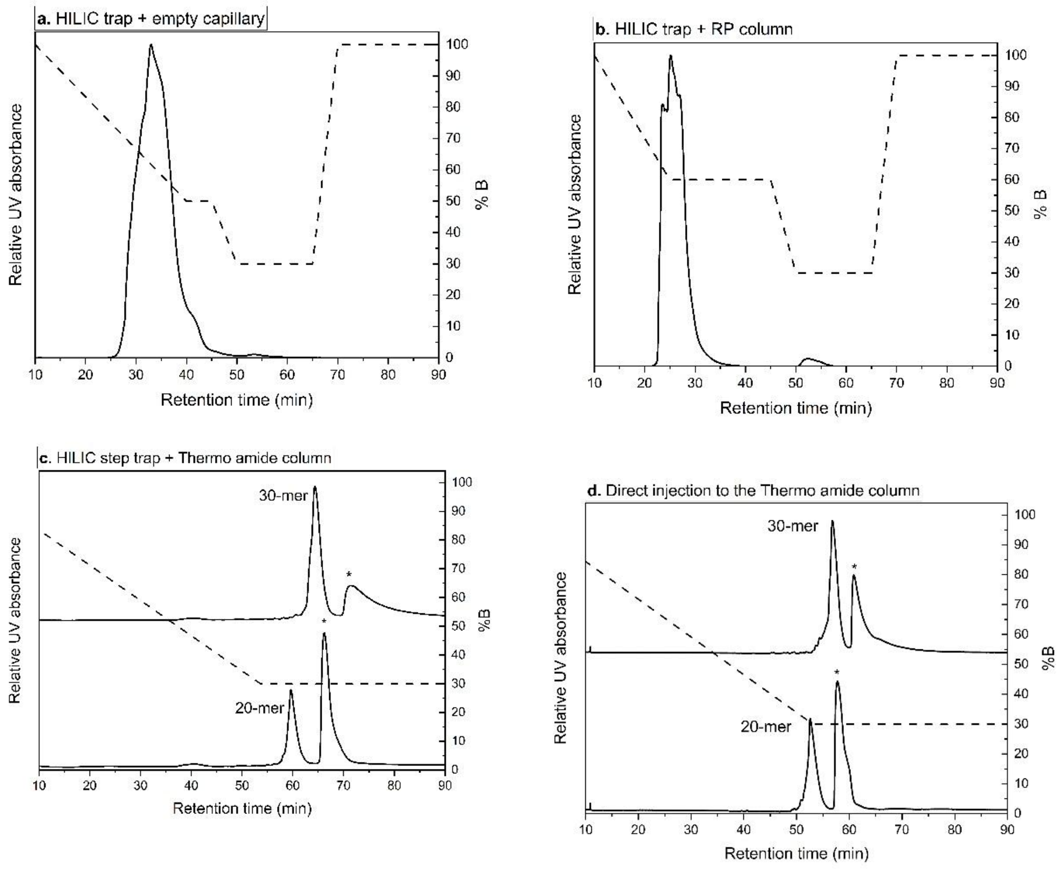

Figure S9 Relative UV absorbance at 260 nm of the mixture of a 20-mer and a 30-mer synthetic RNA oligonucleotides (solid line) that had been (5 min at 10 µL/min) trapped on the HILIC stem trap and eluted onto **a.** an empty capillary (150 mm x 0.075 mm), **b.** a nano-flow RP and **c.** a nano-flow HILIC using the “pre-concentration” injection mode. The analysis was repeated using the “direct” injection mode on the **d.** nano-flow HILIC column. The elution flow rates for all analyses were 0.25 µL/min. The corresponding chromatographic gradients (shown as %B) were also indicated as dashed lines. Impurities were present in these samples as marked by the asterisk (*) signs.

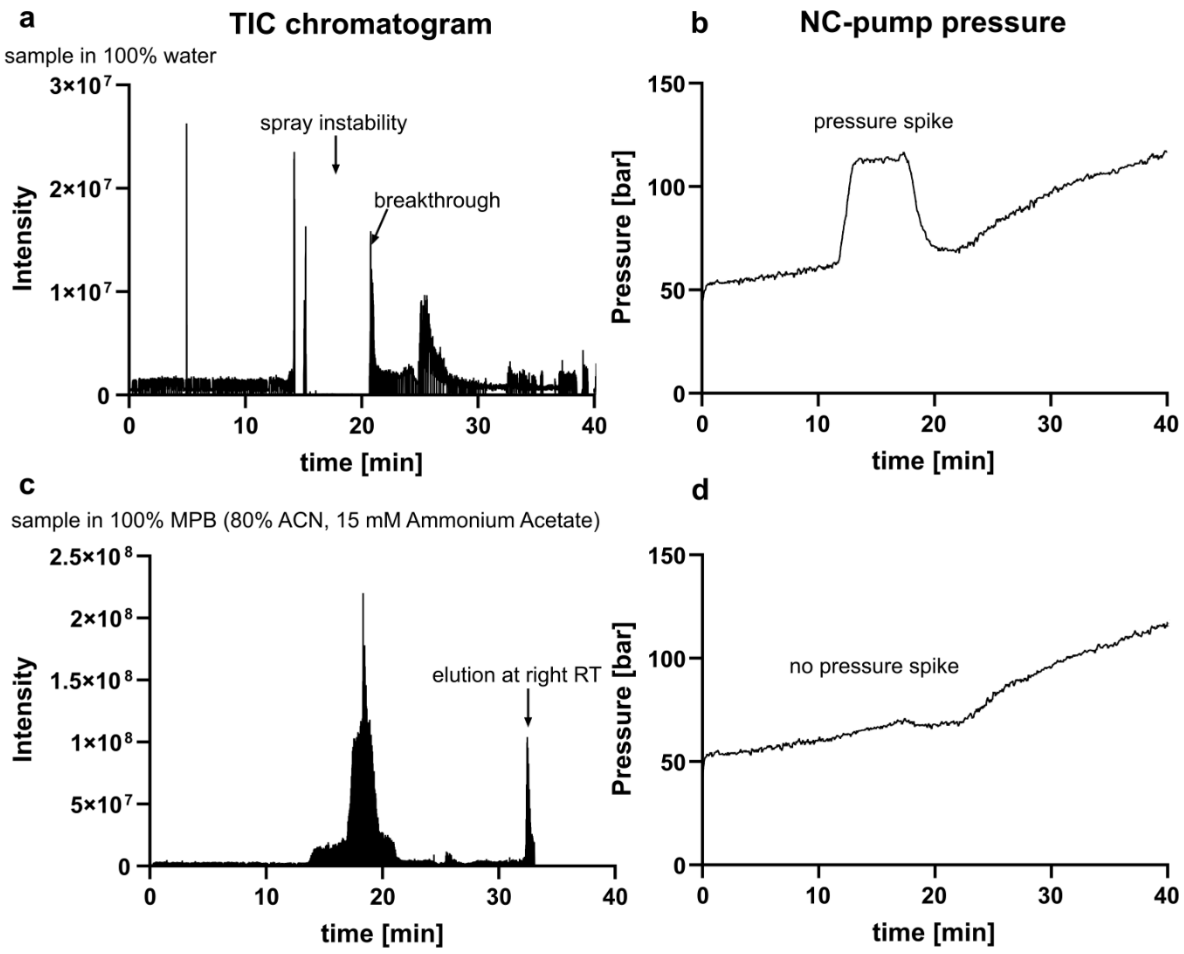

Figure S10. TIC and NC-pump trace while direct injection of **a** and **b** 100% aqueous sample, **c** and **d** sample resuspended in 100% mobile phase B (80% ACN, 15 mM ammonium acetate).

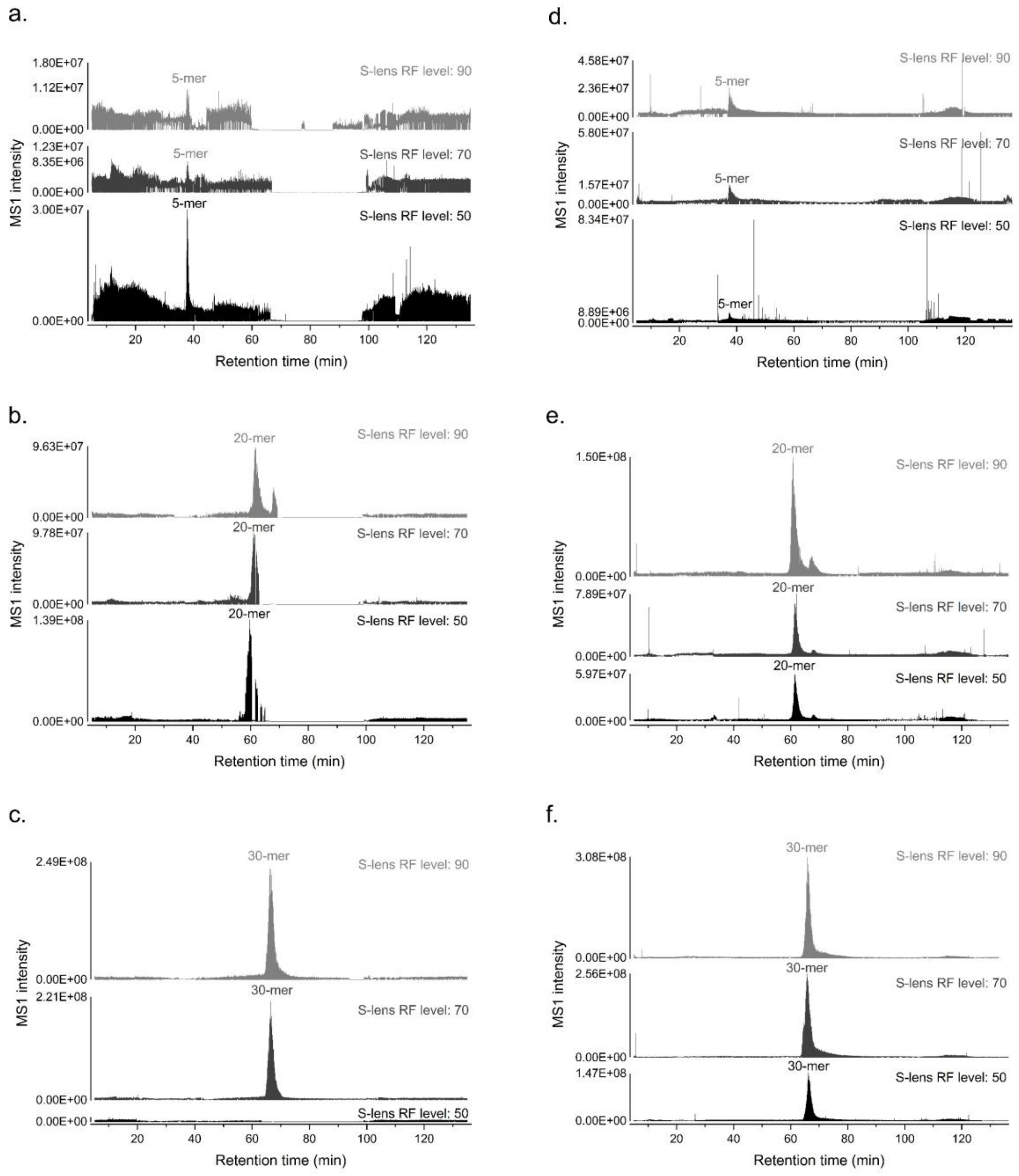

Figure S11 Total ion chromatograms (TICs) of a 5-mer, 20-mer and a 30-mer (synthetic ribonucleotides) under different S-lens RF levels [i.e. 50 (shown in gray), 70 (shown in dark gray), and 90 (shown in black)] analysed under the regular setup (**a-c**) or the infusion setup (d-e).

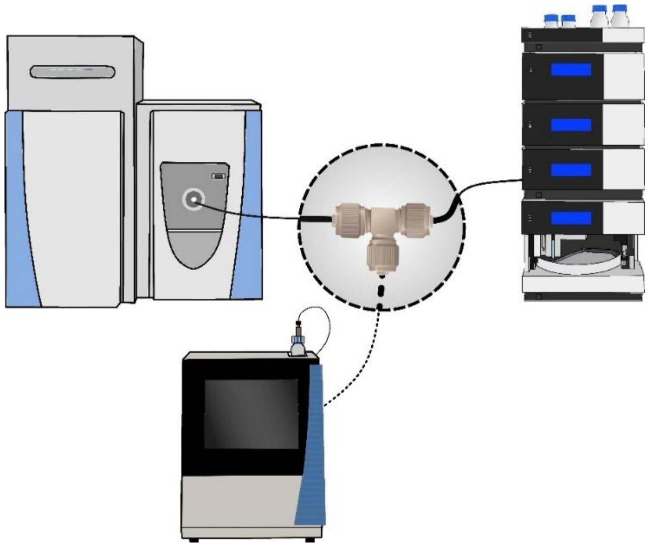

Figure S12. Schematic of the instrumental infusion setup. The infusion flow was indicated by the dashed line.

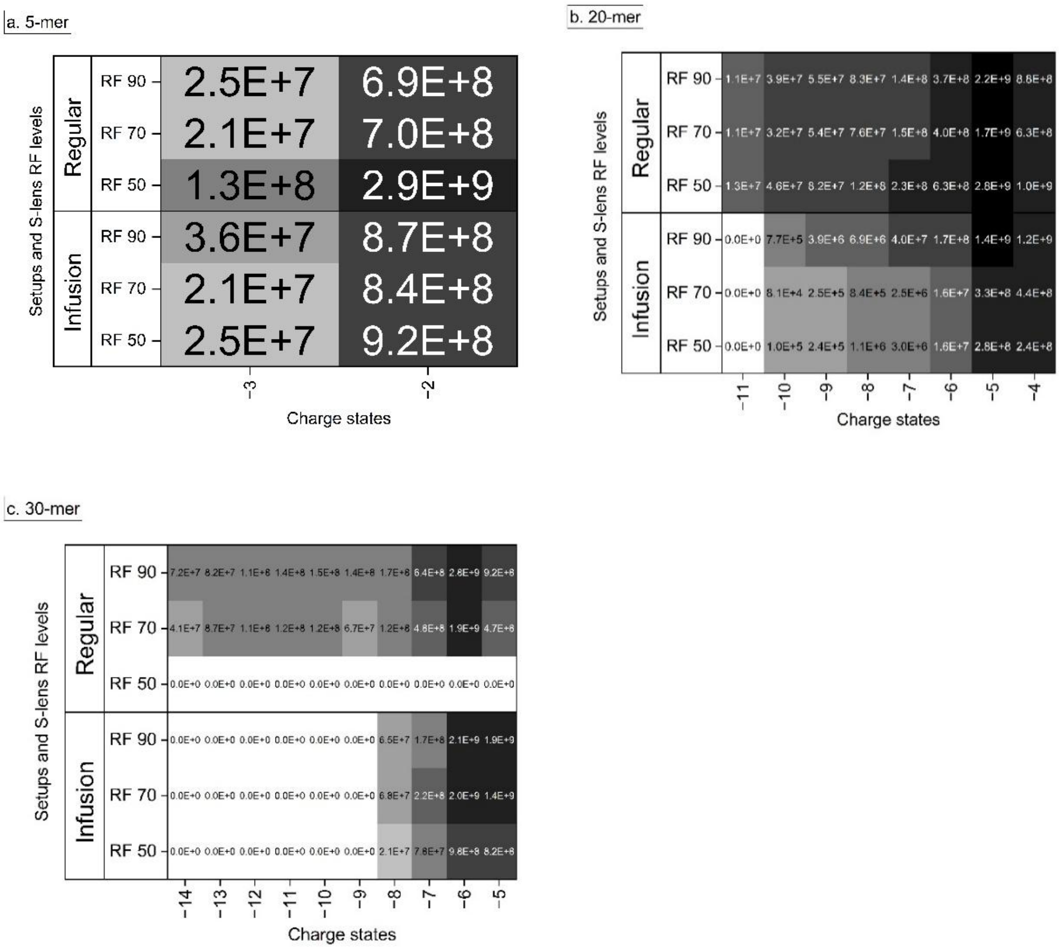

Figure S13 MS[1] signal intensity comparison under different charge states between setups and polarities among **a.** 5-mer, **b.** 20-mer and **c.** 30-mer (synthetic ribonucleotides). The sum of area under curves (AUCs) of the first four isotopic peaks, i.e. [M], [M+1], [M+2], and [M+3], for each charge states were manually integrated in the “skyline” software as the intensity of the corresponding precursor ions.

5-mer	RF 50	RF 70	RF 90
regular	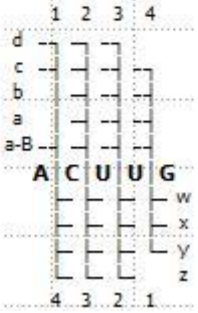	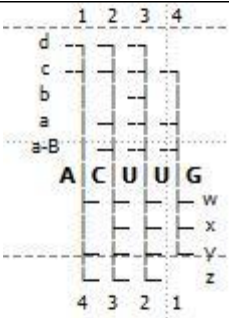	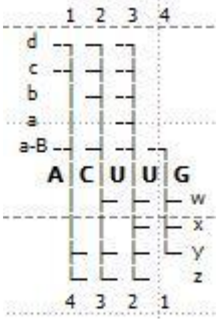
Infusion	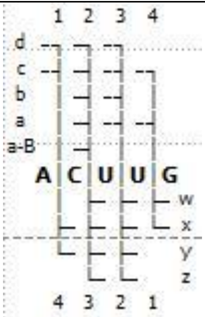	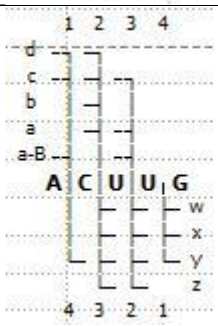	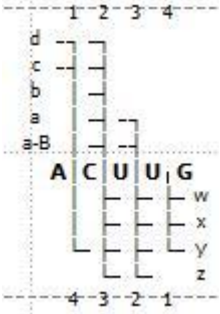
**20-mer**			
regular	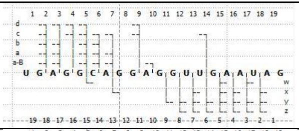	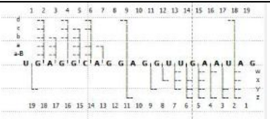	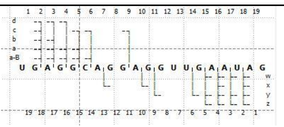
Infusion	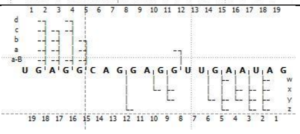	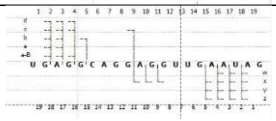	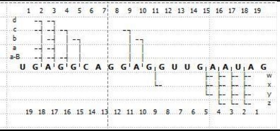
**30-mer**			
regular	N/A	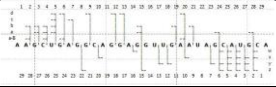	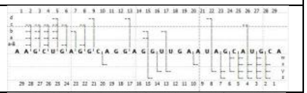
Infusion	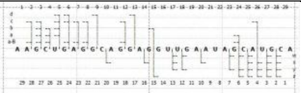	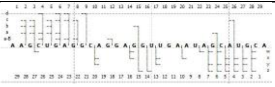	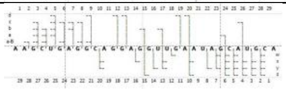

Figure S14. MS^2 sequence coverage comparison between the regular and infusion setup according to NASE analyses. Only the MS^2 evidence with the highest matching score was shown as examples.

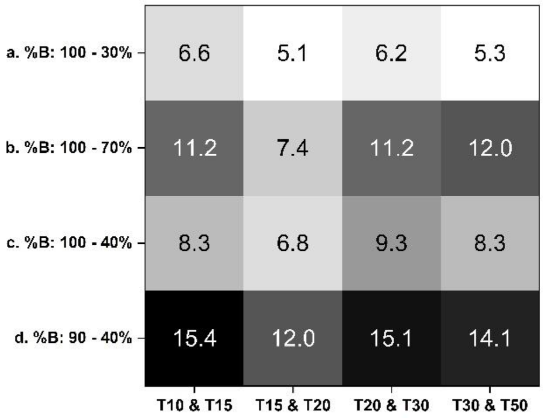

Figure S15. Resolutions of indicated peak pairs. **a.** 0-7 min at 100% B, reaches 30% B at 61 min and lasts until 80 min, equilibrates at 100% B from 90 to 140 min. **b.** 0-5 min at 100% B, reaches 40% B at 100 min and lasts until 120 min, equilibrates at 100% B from 130 to 150 min. **c.** 0-5 min at 100% B, reaches 70% B and 40% B at 100 min and 110 min, respectively. After holding at 40% B till 130 min, equilibrates at 100% B from 140 to 170 min. **d.** 0-5 min at 100% B, reaches 90% B, 70% B, and 40% B at 10 min, 100 min and 150 min, respectively. After holding at 40% B till 170 min, equilibrates at 100% B from 180 to 210 min.

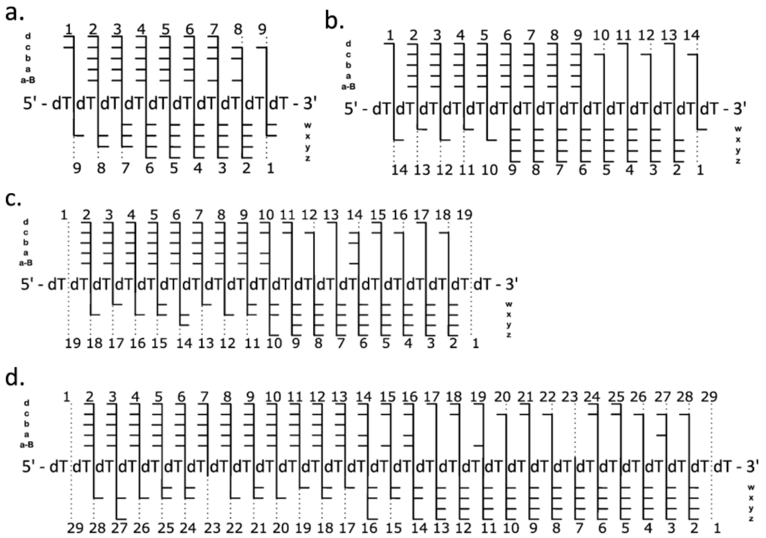

Figure S16. MS^2 fragmented evidence of **a.** dT10, **b.** dT15, **c.** dT20, and **d.** dT30 identified by NASE among all corresponding MS^2 spectra under DDA data acquisition mode from the DNA mixture sample (while the S-lens RF level set at 90). Matched a-B, a, b, c, d, w, x, y, and z ions from the NASE analysis were indicated correspondingly.

tRNA^{Ile} digested by T1 - unspecific cleavage

Observed			Theoretical*						
**m/z**	**Charge**	**Precursor error (|ppm|)**	**Molecular mass**	**Search parameter**	**Oligonucleotide length**	**Oligonucleotide sequence**	**Score**	**cleavage site**	**Retention time (min)**
840,11	-3	2,25	2396,529	T1: 3'-P8-mer		CACCCCUG-P	275,86	G|; G|	24,90
950,46	-3	4,03	2710,725	T1: 3'-P9-mer		UCCACUCAG-P	271,85	G|; G|	25,82
968,12	-2	2,12	1843,170	T1: 3'-P6-mer		UUCAAG-P	161,99	G|; G|	22,76
738,43	-3	2,24	2107,345	T1: 3'-P7-mer		ACCCCUG-P	138,85	G|; G|	23,50
803,10	-2	1,80	1528,975	T1: 3'-P5-mer		CUCAG-P	115,30	G|; G|	23,18
827,12	-2	2,44	1577,025	T1: 3'-P5-mer		AUAAG-P	104,26	G|; G|	22,90
732,76	-3	9,38	2067,219	T1: 3'-P7-mer		CACCCCU-P	83,47	G|; U|	22,88
651,08	-2	9,87	1240,775	T1: 3'-P4-mer		UUAG-P	82,68	G|; G|	21,52
732,77	-3	9,84	2091,345	T1: 3'-P7-mer		CCACUCA-P	80,27	U|; A|	24,59
926,12	-2	3,37	1801,175	T1: 3'-P6-mer		CCACCA-P	72,04	C|	22,82
971,12	-3	9,07	2710,725	T1: 3'-P9-mer		AGUCCACUC-P	69,67	A|; C|	23,44
969,80	-3	0,62	2709,740	T1: 3'-P9-mer		CACCCCUGA-P	69,14	G|; A|	24,08
968,12	-2	8,90	1842,185	T1: 3'-P6-mer		ACUCAG-P	59,79	C|; G|	24,62
965,11	-3	6,81	2734,750	T1: 3'-P9-mer		UCAAGUCCA-P	58,88	U|; A|	24,47
854,76	-3	4,74	2438,525	T1: 3'-P8-mer		UGUAGCUC-P	49,34	U|; C|	24,02
983,77	-3	9,79	2774,775	T1: 3'-P9-mer		CCUGAUAAG-P	47,26	C|; G|	23,98
960,46	-3	0,90	2725,740	T1: 3'-P9-mer		CUCAGGCCC-P	47,21	A|; C|	26,29
841,43	-3	6,46	2397,515	T1: 3'-P8-mer		CCCCUGAU-P	42,95	A|; U|	23,62
855,10	-3	7,08	2420,555	T1: 3'-P8-mer		CCACUCAG-P	40,22	U|; G|	23,82
1271,66	-2	3,74	2396,529	T1: 3'-P8-mer		CACCCCUG-P	37,25	G|; G|	24,89
773,61	-2	1,30	1511,990	T1: 3'-P5-mer		CACCA-P	36,71	C|	22,26
1181,82	-3	7,00	3353,145	T1: 3'-P11-mer		AGUCCACUCAG-P	34,52	A|; G|	22,78
1286,17	-2	6,13	2444,580	T1: 3'-P8-mer		CAAGUCCA-P	34,30	U|; A|	23,27
814,09	-2	1,82	1528,975	T1: 3'-P5-mer		GUCCA-P	33,33	A|; A|	23,84

tRNA^{Ile} digested by RNase 4 - unspecific cleavage

Observed			Theoretical*						
**m/z**	**Charge**	**Precursor error (|ppm|)**	**Molecular mass**	**Search parameter**	**Oligonucleotide length**	**Oligonucleotide sequence**	**Score**	**cleavage site**	**Retention time (min)**
998,64	-2	7,67	1842,255	RNase 4: 2',3'-cP	6-mer	AAGGGU-cP	104,53	U|A; U|G	21,69
665,42	-3	6,76	1842,255	RNase 4: 2',3'-cP	6-mer	AAGGGU-cP	104,21	U|A; U|G	22,16
926,62	-2	8,01	1698,155	RNase 4: 2',3'-cP	6-mer	ACCCCU-cP	86,43	C|A; U|G	22,40
794,10	-2	6,57	1448,995	RNase 4: 2',3'-cP	5-mer	AGCUC-cP	100,53	U|A; C|A	21,74
661,58	-2	4,89	1199,835	RNase 4: 2',3'-cP	4-mer	AGGU-cP	71,84	C|A; U|G	21,14
665,09	-3	9,64	1779,190	RNase 4: 2',3'-cP	6-mer	UCAGGU-cP	8,40	C|U; U|G	20,68
1066,81	-3	0,81	2982,010	RNase 4: 2',3'-cP	10-mer	AGGCCCACCA-cP	178,79	C|A	24,62

tRNA^{Ile} digested by Colicin E5 - unspecific cleavage

Observed			Theoretical*						
**m/z**	**Charge**	**Precursor error (|ppm|)**	**Molecular mass**	**Search parameter**	**Oligonucleotide length**	**Oligonucleotide sequence**	**Score**	**cleavage site**	**Retention time (min)**
971,12	-3	0,92	2915,378	unspecific: 2',3'-cP	9-mer	UAGCUCAGG-cP	254,27	G|U; G|U	23,46
728,09	-4	1,15	2915,378	unspecific: 2',3'-cP	9-mer	UAGCUCAGG-cP	228,61	G|U; G|U	23,71
767,10	-3	7,76	2304,312	unspecific: 2',3'-cP	7-mer	UAGAGCG-cP	182,39	U|U; G|C	22,90
719,42	-3	6,67	2161,266	unspecific: 2',3'-cP	7-mer	UCCACUC-cP	174,46	G|U; C|A	22,83
944,12	-3	0,74	2834,381	unspecific: 2',3'-cP	9-mer	CACCCCUGA-cP	155,03	G|C; A|U	23,82
947,12	-2	6,46	1896,231	unspecific: 2',3'-cP	6-mer	UAGCUC-cP	126,70	G|U; C|A	22,27
868,79	-3	9,58	2609,353	unspecific: 2',3'-cP	8-mer	AGCUCAGG-cP	123,69	U|A; G|U	23,20
665,42	-3	6,21	1999,271	unspecific: 2',3'-cP	6-mer	UAAGGG-cP	123,55	A|U; G|U	22,37
950,46	-3	0,20	2850,376	unspecific: 2',3'-cP	9-mer	CUCAGGCCC-cP	123,21	A|C; C|A	24,01
834,11	-2	5,91	1670,218	unspecific: 2',3'-cP	5-mer	UGAGG-cP	118,99	G|U; G|U	21,92
806,11	-2	5,14	1614,217	unspecific: 2',3'-cP	5-mer	UCAAG-cP	118,85	U|U; G|U	21,61
998,64	-2	6,45	1999,271	unspecific: 2',3'-cP	6-mer	UAAGGG-cP	97,25	A|U; G|U	22,22
959,12	-2	5,74	1920,242	unspecific: 2',3'-cP	6-mer	UUCAAG-cP	83,00	G|U; G|U	21,90
966,63	-2	7,76	1935,253	unspecific: 2',3'-cP	6-mer	CUCAGG-cP	72,88	G|C; G|U	22,40
794,10	-2	5,03	1590,206	unspecific: 2',3'-cP	5-mer	AGCUC-cP	68,92	U|A; C|A	21,96
650,08	-2	9,61	1301,160	unspecific: 2',3'-cP	4-mer	UCGG-cP	68,42	G|U; G|U	20,92
661,59	-2	8,98	1325,171	unspecific: 2',3'-cP	4-mer	UGAG-cP	56,07	G|U; G|G	20,82
834,11	-3	3,41	2505,329	unspecific: 2',3'-cP	8-mer	CACCCCUG-cP	55,32	G|C; G|A	23,72
950,46	-3	3,45	2853,375	unspecific: 3'-P	9-mer	UCCACUCAG-P	237,08	G|U; G|G	24,01
840,11	-3	3,12	2523,339	unspecific: 3'-P	8-mer	CACCCCUG-P	211,36	G|C; G|A	23,92
968,13	-2	7,10	1938,252	unspecific: 3'-P	6-mer	UUCAAG-P	111,99	G|U; G|U	22,74
803,11	-2	7,59	1608,216	unspecific: 3'-P	5-mer	CUCAG-P	104,01	G|C; G|G	22,59
827,12	-2	6,93	1656,239	unspecific: 3'-P	5-mer	AUAAG-P	100,60	G|A; G|G	22,36
978,12	-3	6,31	2933,388	unspecific: 3'-P	9-mer	UAGCUCAGG-P	69,43	G|U; G|U	23,29
971,46	-3	1,12	2917,393	unspecific: 3'-P	9-mer	CCUGAUAAG-P	59,23	C|C; G|G	25,66
651,08	-2	4,95	1304,159	unspecific: 3'-P	4-mer	UUAG-P	58,89	G|U; G|A	21,60
950,47	-3	7,84	2852,391	unspecific: 3'-P	9-mer	CACCCCUGA-P	56,34	G|C; A|U	24,53
863,10	-3	9,26	2588,341	unspecific: 3'-P	8-mer	UAGCUCAG-P	50,27	G|U; G|G	22,99

Figure S17 Comparison of RNAse 4, colicin E5 and Rnase T1 digested unmodified RNA *E. c.* tRNA$^{Ile}_{GAU}$) using updated NASE. Table of found fragments for unspecific digestion for RNase T1, RNase 4 and Colicin E5. cP = 3’ cyclic phosphate, P = 3’ phosphate and OH = 3’ hydroxyl. In the column “cleavage site”, the 5’-cleavage site and 3’-cleavage sites are separated by semicolon (;).

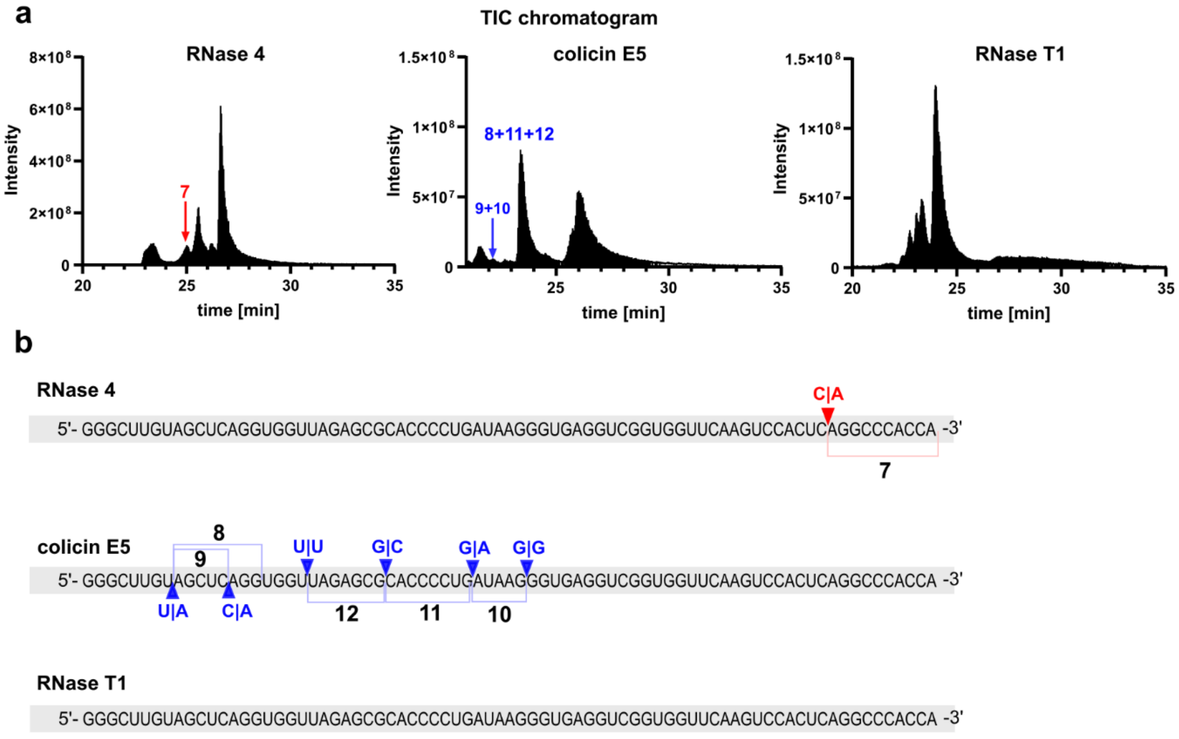

Figure S18 Unspecific cleavage products found in RNase T1, RNase 4 and colicin E5 digested unmodified RNA (*E. c.* tRNA$^{Ile}_{GAU}$). **a** TIC with arrows indicating RT of found fragments. **b** Assigned fragments and off-target cleavage sites in the sequence of *E. c.* tRNA$^{Ile}_{GAU}$. Red: RNase 4; blue: colicin E5; grey: RNase T1.

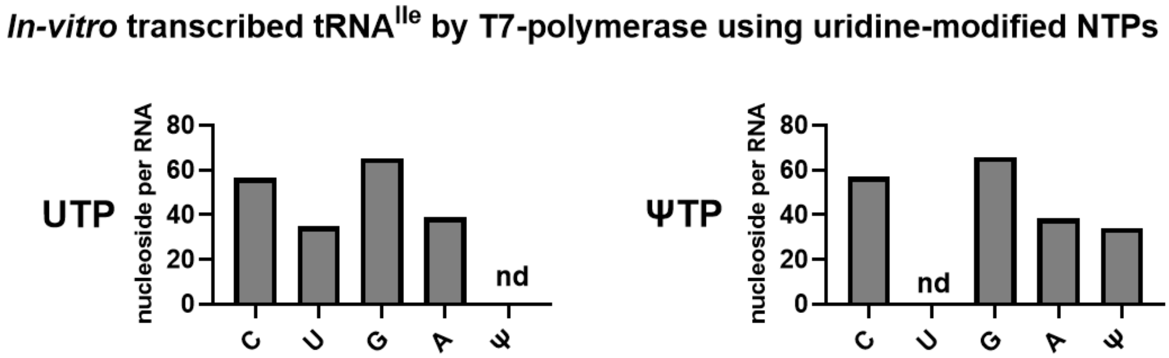

Figure S19 LC-MS/MS Quantification of nucleosides in tRNA^{Ile} using either UTP or ΨTP during transcription. Oligonucleotide MS analysis is shown in Figure 6.

tRNA^{Phe} + RNase T1

Observed								
**m/z**	**Charge**	**Precursor error**	**Search parameter**	**Oligonucleotide length**	**Oligonucleotide sequence**	**Score**	**cleavage site**	**Retention time (min)**
1400,54	-3	1,55	T1: 3'-P	12-mer	A[Cm]U[Gm]AA[yW]AY[m5C]UG-P	73,28	G|	23,4
1050,16	-4	4,82	T1: 3'-P	12-mer	A[Cm]U[Gm]AA[yW]AY[m5C]UG-P	62,99	G|	23,45

tRNA^{Phe} + RNase 4

Observed								
**m/z**	**Charge**	**Precursor error**	**Search parameter**	**Oligonucleotide length**	**Oligonucleotide sequence**	**Score**	**cleavage site**	**Retention time (min)**
954,50	-3	4,57	RNase 4: 2',3'-cP	8-mer	[Gm]AA[yW]AY[m5C]U-cP	97,18	U|G_m; U|G	22,84
983,12	-3	8,70	RNase 4: 2',3'-cP	9-mer	A[m2G]CUCAGDD-cP	76,83	U|A; D|G	23,11
1378,66	-4	0,41	RNase 4: 2',3'-cP	17-mer	GCGGAUUUA[m2G]CUCAGDD-cP	12,68	D|G	24,94
985,81	-3	9,08	RNase 4: 2',3'-cP	9-mer	GGAG[m7G]UC[m5C]U-cP	115,69	U|G; U|G	24,71
1338,67	-4	1,87	RNase 4: 2',3'-cP	16-mer	GGGAGAGC[m2,2G]CCAGA[Cm]U-cP	94,34	D|G; U|G_m	25,30
959,80	-3	8,92	unspecific cleavage: 2',3'-cP	9-mer	CCACAGAAU-cP	130,95	U|A	23,59
1189,81	-3	6,66	unspecific cleavage: 2',3'-cP	11-mer	G[m5U]YCG[m1A]UCCAC-cP	80,39	U|C; U|U	23,97

tRNA^{Phe} + Colicin E5

Observed								
**m/z**	**Charge**	**Precursor error**	**Search parameter**	**Oligonucleotide length**	**Oligonucleotide sequence**	**Score**	**cleavage site**	**Retention time (min)**
1454,41	-5	6,67	Colicin E5: 2',3'-cP	23-mer	[m5U]YCG[m1A]UCCACAGAAUUCGCACCA	101,56	G|U; G|U	25,63
1297,85	-2	14,00	Colicin E5: 2',3'-cP	5-mer	UC[m5C]UG-cP	96,66	G|U; G|U	21,63
754,11	-3	9,38	Colicin E5: 2',3'-cP	7-mer	UC[m5C]UGUG-cP	29,12	G|U; G|U	22,70
1055,81	-3	2,77	unspecific cleavage: 2',3'-cP	10-mer	[m5U]YCG[m1A]UCCAC-cP	182,82	G|m^5U; C|A	23,71
863,78	-3	4,25	unspecific cleavage: 2',3'-cP	8-mer	AGAAUUCG-cP	66,18	A|G; G|C	23,08
965,79	-3	1,97	unspecific cleavage: 2',3'-cP	9-mer	AGAAUUCGC-cP	69,12	A|G; C|A	23,46
957,78	-3	7,40	unspecific cleavage: 2',3'-cP	9-mer	AUUUA[m2G]CUC-cP	52,89	G|A; C|A	23,34
965,79	-3	2,54	unspecific cleavage: 2',3'-cP	9-mer	CAGAAUUCG-cP	49,85	A|C; G|C	23,64
1067,14	-3	2,45	unspecific cleavage: 2',3'-cP	10-mer	CAGAAUUCGC-cP	72,68	A|C; C|A	23,76
997,81	-3	5,34	unspecific cleavage: 2',3'-cP	9-mer	DGGGAGAGC-cP	131,54	D|D; C|$m^2{}_2$G	23,77
868,11	-3	6,43	unspecific cleavage: 2',3'-cP	8-mer	G[m1A]UCCACA-cP	89,88	C|G; A|G	23,17
973,79	-3	7,40	unspecific cleavage: 2',3'-cP	9-mer	GAAUUCGCA-cP	59,73	A|G; A|C	23,33
1081,15	-3	7,56	unspecific cleavage: 2',3'-cP	10-mer	GAAUUCGCAC-cP	65,74	A|G; C|C	23,81
860,45	-3	2,99	unspecific cleavage: 2',3'-cP	8-mer	UA[m2G]CUCAG-cP	211,38	U|U; G|D	22,96
1062,47	-3	1,15	unspecific cleavage: 2',3'-cP	10-mer	UCCACAGAAU-cP	73,30	m^1A|U; U|U	23,57
1379,21	-3	2,50	unspecific cleavage: 2',3'-cP	8-mer	UCGCACCA	194,52	U|U	23,18

Figure S20 Comparison of RNase 4, colicin E5 and RNase T1 digested native RNA *S. c.* tRNA$^{Phe}{}_{GmAA}$) using updated NASE. Table of found fragments for RNase T1, RNase 4 and Colicin E5. cP = 3' cyclic phosphate, P = 3' phosphate and OH = 3' hydroxyl. In the column "cleavage site", the 5'-cleavage site and 3'-cleavage sites are separated by semicolon (;).

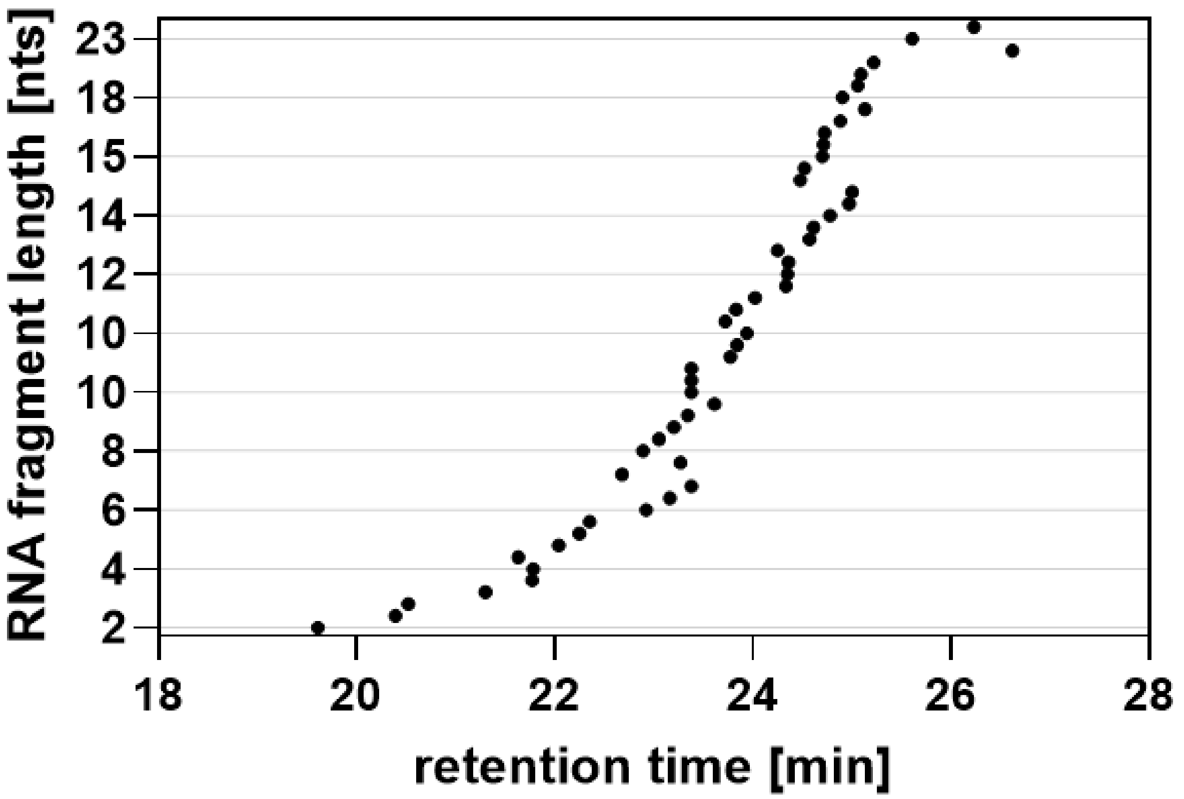

Figure S21 Lengths of RNA fragments detected from FLuc-mRNA after RNase T1, RNase 4 or colicin E5 cleavage plotted over retention time.

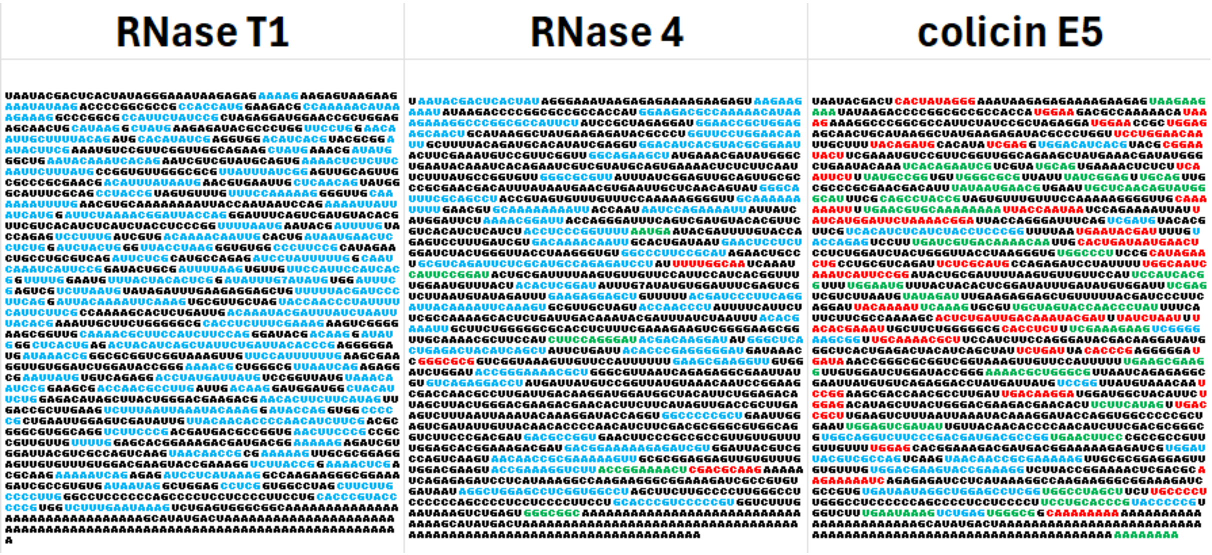

Figure S22 Comparison of RNase 4, colicin E5 and RNase T1 digested FLuc-mRNA from NIST using updated NASE. Blue fragments: Expected cleavage site on both ends, green fragments: One expected and one unexpected cleavage site and red fragments: Both cleavage sites are unexpected.

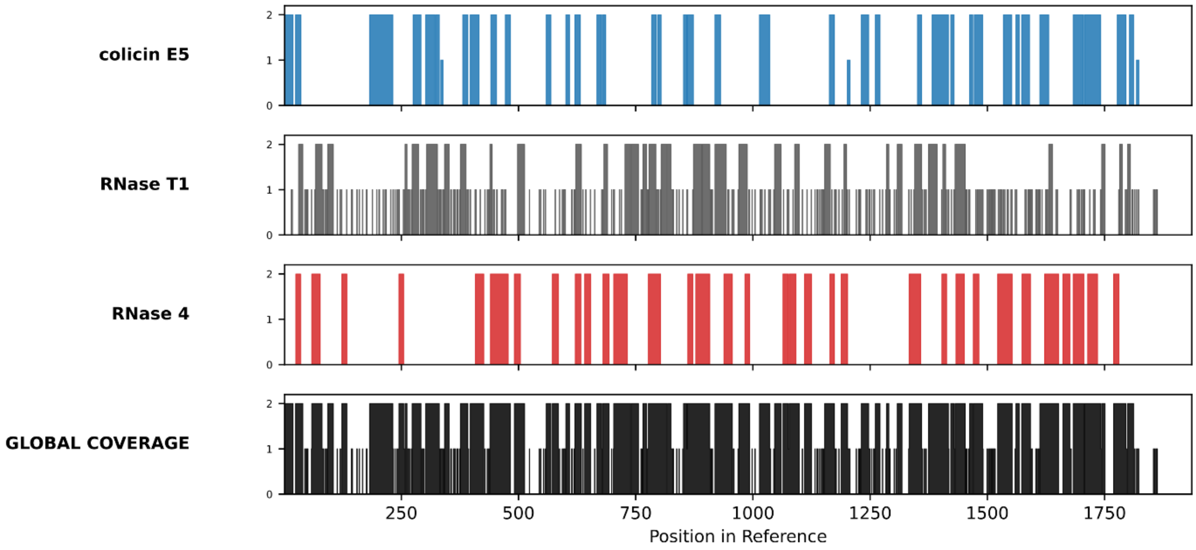

Figure S23 Sequence coverage maps obtained from Firefly luciferase (FLuc) mRNA which is digested with RNase 4, RNase T1 and colicin E5, calculated with BPF (ThermoFisher BioPharma Finder). Global coverage shows combined coverage from all 3 nucleases.

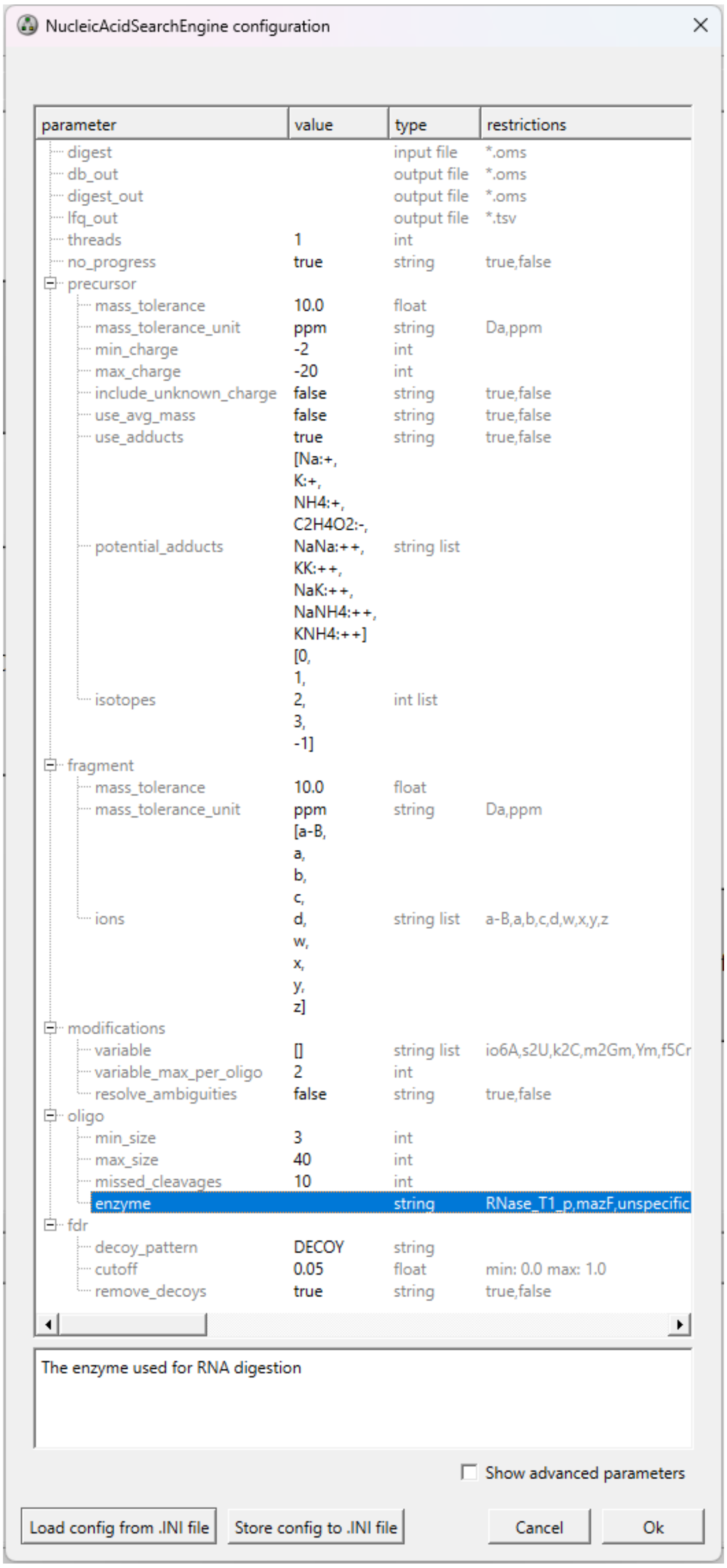

Figure S24 NASE configuration for the data analysis of IVT and biological RNA samples. The right nuclease is chosen for the row “enzyme”, which is highlighted in blue.

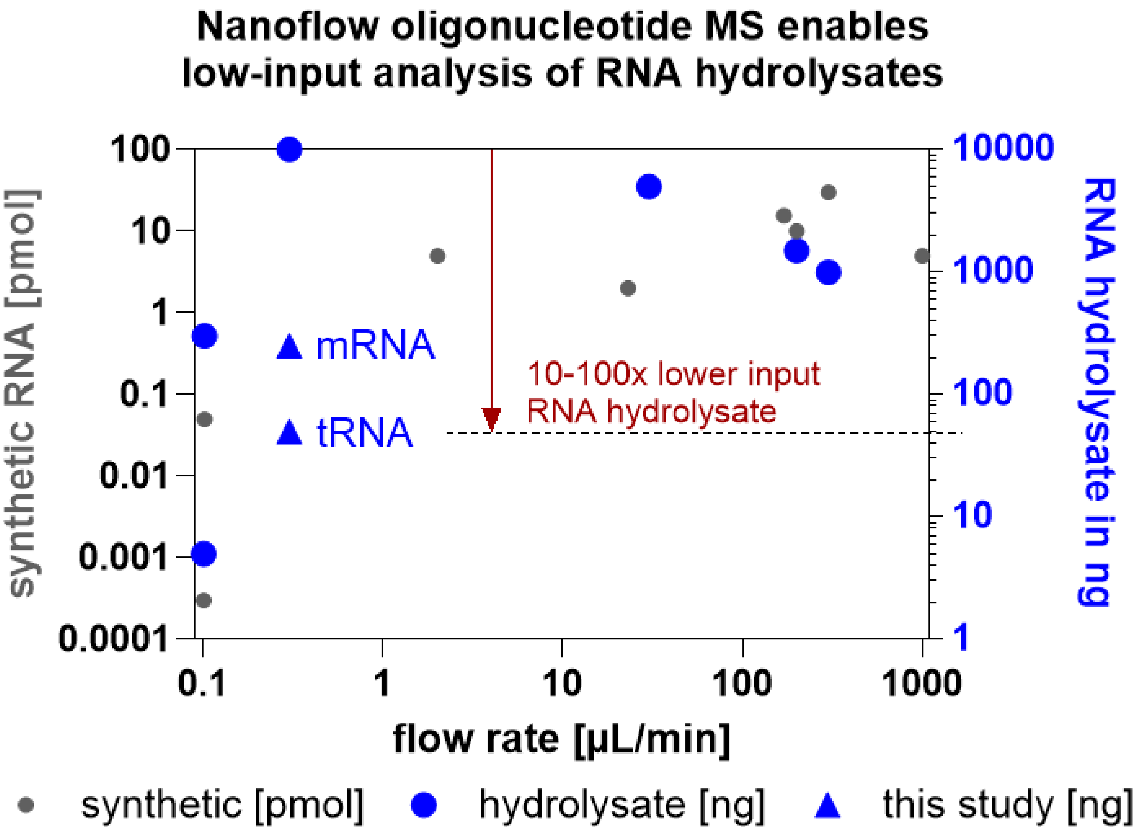

Figure S25 Comparison of this study in terms of required input material over flow rate. Cited studies are summarized in Table S2.

Supplementary files

NASE_Master_Browser.html and ThermoBPF_Master_Browser.html:

interactive HTML file for a detailed exploration of the mRNA sequence coverage data generated by different nucleases. Users can interactively toggle the visibility of individual RNases or view them in combination by using the legends. By placing the cursor over the coverage bars, specific information for each nucleotide position is displayed. The height of the bars and the corresponding value indicates the mapping status: 0 (No Coverage): The specific position was not covered by any detected fragments for the selected RNase(s); 1 (Non-Unique Coverage): The position is covered by fragments that are also generated or shared by other RNases within the dataset; 2 (Unique Coverage): The position is covered by a fragment that is uniquely produced by the selected RNase, providing specific sequence information that is not redundant with other nucleases.

Supplementary References

[1] R. A. Deshpande and V. Shankar, 'Ribonucleases from T2 Family', Crit. Rev. Microbiol., vol. 28, no. 2, pp. 79–122, 2002, doi: 10.1080/1040-840291046704.

[2] K. Höschler, H. Hoier, B. Hubner, W. Saenger, P. Orth, and U. Hahn, ‘Structural analysis of an RNase T1 variant with an altered guanine binding segment’, J. Mol. Biol., vol. 294, no. 5, pp. 1231–1238, Dec. 1999, doi: 10.1006/JMBI.1999.3324.

[3] S. Noguchi, Y. Satow, T. Uchida, C. Sasaki, and T. Matsuzaki, ‘Crystal Structure of Ustilago sphaerogena Ribonuclease U2 at 1.8 Å Resolution’, Biochemistry, vol. 34, no. 47, pp. 15583–15591, 1995, doi: 10.1021/BI00047A025/ASSET/BI00047A025.FP.PNG_V03.

[4] N. L. Mironova, D. V. Pyshnyi, D. V. Shtadler, A. A. Fedorova, V. V. Vlassov, and M. A. Zenkova, ‘RNase T1 mimicking artificial ribonuclease’, Nucleic Acids Res., vol. 35, no. 7, pp. 2356–2367, Apr. 2007, doi: 10.1093/NAR/GKM143.

[5] T. Jiang et al., ‘Oligonucleotide Sequence Mapping of Large Therapeutic mRNAs via Parallel Ribonuclease Digestions and LC-MS/MS’, Anal. Chem., vol. 91, no. 13, pp. 8500–8506, Jul. 2019, doi: 10.1021/ACS.ANALCHEM.9B01664/SUPPL_FILE/AC9B01664_SI_001.PDF.

[6] E. J. Wolf et al., ‘Human RNase 4 improves mRNA sequence characterization by LC–MS/MS’, Nucleic Acids Res., vol. 50, no. 18, p. e106, Oct. 2022, doi: 10.1093/NAR/GKAC632.

[7] B. Solivio, N. Yu, B. Addepalli, and P. A. Limbach, ‘Improving RNA modification mapping sequence coverage by LC-MS through a nonspecific RNase U2-E49A mutant’, Anal. Chim. Acta, vol. 1036, pp. 73–79, Dec. 2018, doi: 10.1016/J.ACA.2018.08.012.

[8] C. Herbert, S. Valesyan, J. Kist, and P. A. Limbach, ‘Analysis of RNA and its Modifications’, Annu. Rev. Anal. Chem. (Palo Alto Calif), vol. 17, no. 1, p. 47, Jul. 2024, doi: 10.1146/ANNUREV-ANCHEM-061622-125954.

[9] J. Mengel-Jørgensen and F. Kirpekar, ‘Detection of pseudouridine and other modifications in tRNA by cyanoethylation and MALDI mass spectrometry’, Nucleic Acids Res., vol. 30, no. 23, p. e135, 2002, doi: 10.1093/NAR/GNF135.

[10] R. Hartmer et al., ‘RNase T1 mediated base-specific cleavage and MALDI-TOF MS for high-throughput comparative sequence analysis’, Nucleic Acids Res., vol. 31, no. 9, p. e47, 2003, doi: 10.1093/NAR/GNG047.

[11] T. Arima, T. Uchida, and F. Egami, ‘Studies on extracellular ribonucleases of Ustilago sphaerogena. Characterization of substrate specificity with special reference to purine-specific ribonucleases’, Biochemical Journal, vol. 106, no. 3, p. 609, 1968, doi: 10.1042/bj1060609.

[12] P. Thakur, S. Abernathy, P. A. Limbach, and B. Addepalli, ‘Locating chemical modifications in RNA sequences through ribonucleases and LC-MS based analysis’, Methods Enzymol., vol. 658, p. 1, Jan. 2021, doi: 10.1016/BS.MIE.2021.06.023.

[13] N. Luhtala and R. Parker, ‘T2 Family Ribonucleases: Ancient enzymes with diverse roles’, Trends Biochem. Sci., vol. 35, no. 5, p. 253, May 2010, doi: 10.1016/J.TIBS.2010.02.002.

[14] W. Greulich et al., ‘TLR8 is a sensor of RNase T2 degradation products’, Cell, vol. 179, no. 6, p. 1264, Nov. 2019, doi: 10.1016/J.CELL.2019.11.001.

[15] M. Irie and K. Ohgi, ‘Ribonuclease T2’, Methods Enzymol., vol. 341, pp. 42–55, Jan. 2001, doi: 10.1016/S0076-6879(01)41144-X.

[16] A. Minami et al., ‘The ribonuclease RNase T2 mediates selective autophagy of ribosomes induced by starvation in Saccharomyces cerevisiae’, J. Biol. Chem., vol. 301, no. 6, p. 108554, Jun. 2025, doi: 10.1016/J.JBC.2025.108554.

[17] A. Suzuki et al., ‘Crystal structures of the ribonuclease MC1 from bitter gourd seeds, complexed with 2’-UMP or 3’-UMP, reveal structural basis for uridine specificity’, Biochem. Biophys. Res. Commun., vol. 275, no. 2, pp. 572–576, Aug. 2000, doi: 10.1006/bbrc.2000.3318.

[18] P. Thakur, J. Atway, P. A. Limbach, and B. Addepalli, 'RNA Cleavage Properties of Nucleobase-Specific RNase MC1 and Cusativin Are Determined by the Dinucleotide-Binding Interactions in the Enzyme-Active Site', Int. J. Mol. Sci., vol. 23, no. 13, p. 7021, Jul. 2022, doi: 10.3390/ijms23137021.
[19] B. Addepalli, N. P. Lesner, and P. A. Limbach, 'Detection of RNA nucleoside modifications with the uridine-specific ribonuclease MC1 from Momordica charantia', RNA, vol. 21, no. 10, p. 1746, Oct. 2015, doi: 10.1261/RNA.052472.115.
[20] B. Addepalli, S. Venus, P. Thakur, and P. A. Limbach, 'Novel ribonuclease activity of cusativin from Cucumis sativus for mapping nucleoside modifications in RNA', Anal. Bioanal. Chem., vol. 409, no. 24, p. 5645, Sep. 2017, doi: 10.1007/S00216-017-0500-X.
[21] J. F. Shaffer et al., 'Epididymis-specific RNase A family genes regulate fertility and small RNA processing', J. Biol. Chem., vol. 300, no. 12, p. 107933, Dec. 2024, doi: 10.1016/J.JBC.2024.107933.
[22] H. F. Rosenberg, 'RNase A Ribonucleases and Host Defense: an Evolving Story', J. Leukoc. Biol., vol. 83, no. 5, p. 1079, May 2008, doi: 10.1189/JLB.1107725.
[23] S. Sato and S. Takenaka, 'Highly Sensitive Nuclease Assays Based on Chemically Modified DNA or RNA', Sensors 2014, Vol. 14, Pages 12437-12450, vol. 14, no. 7, pp. 12437–12450, Jul. 2014, doi: 10.3390/S140712437.
[24] A. Lechner, P. Wolff, E. Leize-Wagner, and Y. N. François, 'Characterization of Post-Transcriptional RNA Modifications by Sheathless Capillary Electrophoresis-High Resolution Mass Spectrometry', Anal. Chem., vol. 92, no. 10, pp. 7363–7370, May 2020, doi: 10.1021/ACS.ANALCHEM.0C01345/ASSET/IMAGES/LARGE/AC0C01345_0005.JPEG.
[25] J. M. Messmore, D. N. Fuchs, and R. T. Raines, 'Ribonuclease A: Revealing Structure–Function Relationships with Semisynthesis', J. Am. Chem. Soc., vol. 117, no. 31, p. 8057, 1995, doi: 10.1021/ja00136a001.
[26] Y. Zhang, J. Zhang, H. Hara, I. Kato, and M. Inouye, 'Insights into the mRNA Cleavage Mechanism by MazF, an mRNA Interferase', Journal of Biological Chemistry, vol. 280, no. 5, pp. 3143–3150, Feb. 2005, doi: 10.1074/JBC.M411811200.
[27] P. Thakur, M. Estevez, P. A. Lobue, P. A. Limbach, and B. Addepalli, 'Improved RNA modification mapping of cellular non-coding RNAs using C- and U-specific RNases', Analyst, vol. 145, no. 3, p. 816, Feb. 2020, doi: 10.1039/C9AN02111F.
[28] S. Fleurier and I. Matic, 'MazF endoribonuclease promotes resolution of transcription–replication conflicts at ribosomal RNA genes in Escherichia coli', Nucleic Acids Res., vol. 53, no. 19, p. gkaf1034, Oct. 2025, doi: 10.1093/NAR/GKAF1034.
[29] D. K. Simanshu, Y. Yamaguchi, J. H. Park, M. Inouye, and D. J. Patel, 'Structural Basis of mRNA Recognition and Cleavage by Toxin MazF and Its Regulation by Antitoxin MazE in Bacillus subtilis', Mol. Cell, vol. 52, no. 3, p. 447, Nov. 2013, doi: 10.1016/J.MOLCEL.2013.09.006.
[30] S. Yajima, S. Inoue, T. Ogawa, T. Nonaka, K. Ohsawa, and H. Masaki, 'Structural basis for sequence-dependent recognition of colicin E5 tRNase by mimicking the mRNA–tRNA interaction', Nucleic Acids Res., vol. 34, no. 21, p. 6074, Dec. 2006, doi: 10.1093/NAR/GKL729.
[31] Y. Zhang, J. Zhang, K. P. Hoeflich, M. Ikura, G. Qing, and M. Inouye, 'MazF Cleaves Cellular mRNAs Specifically at ACA to Block Protein Synthesis in Escherichia coli', Mol. Cell, vol. 12, no. 4, pp. 913–923, Oct. 2003, doi: 10.1016/S1097-2765(03)00402-7.

[32] G. Han, Q. Lin, J. Yi, Q. Lyu, Q. Ma, and L. Qiao, 'MazF-rolling circle amplification combined MALDI-TOF MS for site-specific detection of N6-methyladenosine RNA', Anal. Chim. Acta, vol. 1303, p. 342532, May 2024, doi: 10.1016/J.ACA.2024.342532.
[33] T. Ogawa, S. Inoue, S. Yajima, M. Hidaka, and H. Masaki, 'Sequence-specific recognition of colicin E5, a tRNA-targeting ribonuclease', Nucleic Acids Res., vol. 34, no. 21, pp. 6065–6073, Nov. 2006, doi: 10.1093/NAR/GKL629.
[34] G. N. Yeşiltaç-Tosun, 'Chemical and Enzymatic Strategies for RNA Modification Analysis by Oligonucleotide Mass Speceader', 2025.
[35] A. Kouvela, A. Zaravinos, and V. Stamatopoulou, 'Adaptor Molecules Epitranscriptome Reprograms Bacterial Pathogenicity', Int. J. Mol. Sci., vol. 22, no. 16, p. 8409, Aug. 2021, doi: 10.3390/IJMS22168409.
[36] Guilherme J. Guimaraes, Jack G. Saad, Vidya Annavarapu, and Michael G. Bartlett. Journal of the American Society for Mass Spectrometry 2023 34 (12), 2691-2699 DOI: 10.1021/jasms.3c00264
[37] Gong L, McCullagh JS. Comparing ion-pairing reagents and sample dissolution solvents for ion-pairing reversed-phase liquid chromatography/electrospray ionization mass spectrometry analysis of oligonucleotides. Rapid Commun Mass Spectrom. 2014 Feb 28;28(4):339-50. doi: 10.1002/rcm.6773. PMID: 24395501.
[38] McGinnis, A.C., Grubb, E.C. and Bartlett, M.G. (2013), Systematic optimization of ion-pairing agents and hexafluoroisopropanol for enhanced electrospray ionization mass spectrometry of oligonucleotides. Rapid Commun. Mass Spectrom., 27: 2655-2664. https://doi.org/10.1002/rcm.6733
[39] Ning Li, N.M. El Zahar, Jack G. Saad, Erik R.E. van der Hage, Michael G. Bartlett, Alkylamine ion-pairing reagents and the chromatographic separation of oligonucleotides, Journal of Chromatography A, Volume 1580, 2018, Pages 110-119, ISSN 0021-9673, https://doi.org/10.1016/j.chroma.2018.10.040.
[40] Honorine Lardeux, Szabolcs Fekete, Matthew Lauber, Valentina D'Atri, and Davy Guillarme. Analytical Chemistry 2023 95 (27), 10448-10456 DOI: 10.1021/acs.analchem.3c01934
[41] Full-Range Profiling of tRNA Modifications Using LC–MS/MS at Single-Base Resolution through a Site-Specific Cleavage Strategy. Tong-Meng Yan, Yu Pan, Meng-Lan Yu, Kua Hu, Kai-Yue Cao, and Zhi-Hong Jiang. Analytical Chemistry 2021 93 (3), 1423-1432 DOI: 10.1021/acs.analchem.0c03307
[42] Hermon, S.J., Sennikova, A. & Becker, S. Quantitative detection of pseudouridine in RNA by mass spectrometry. Sci Rep 14, 27564 (2024). https://doi.org/10.1038/s41598-024-78734-3
[43] Erb R, Oberacher H. Comparison of mobile-phase systems commonly applied in liquid chromatography-mass spectrometry of nucleic acids. Electrophoresis. 2014 May;35(9):1226-35. doi: 10.1002/elps.201300269. Epub 2013 Nov 8. PMID: 24123202.
[44] Fountain KJ, Gilar M, Gebler JC. Analysis of native and chemically modified oligonucleotides by tandem ion-pair reversed-phase high-performance liquid chromatography/electrospray ionization mass spectrometry. Rapid Commun Mass Spectrom. 2003;17(7):646-53. doi: 10.1002/rcm.959. PMID: 12661016.
[45] Enhanced Detection of Post-Transcriptional Modifications Using a Mass-Exclusion List Strategy for RNA Modification Mapping by LC-MS/MS. Analytical Chemistry, 87, 8433-8440
[46] Masato Taoka, Yoshio Yamauchi, Yuko Nobe, Shunpei Masaki, Hiroshi Nakayama, Hideaki Ishikawa, Nobuhiro Takahashi, Toshiaki Isobe, An analytical platform for mass spectrometry-based identification and chemical analysis of RNA in ribonucleoprotein complexes, Nucleic Acids Research, Volume 37, Issue 21, 1 November 2009, Page e140, https://doi.org/10.1093/nar/gkp732

[47] Nakayama, H., Yamauchi, Y., Taoka, M. & Isobe, T. (2015). Direct Identification of Human Cellular MicroRNAs by Nanoflow Liquid Chromatography–High-Resolution Tandem Mass Spectrometry and Database Searching. Analytical Chemistry, 87(5), 2884–2891. https://doi.org/10.1021/ac504378s
[48] Nakayama, H., Yamauchi, Y., Nobe, Y., Sato, K., Takahashi, N., Shalev-Benami, M., Isobe, T. and Taoka, M. (2019) Method for Direct Mass-Spectrometry-Based Identification of Monomethylated RNA Nucleoside Positional Isomers and Its Application to the Analysis of Leishmania rRNA. Analytical Chemistry, 91, 15634-15643.
[49] Holvec, S., Barchet, C., Lechner, A. et al. The structure of the human 80S ribosome at 1.9 Å resolution reveals the molecular role of chemical modifications and ions in RNA. Nat Struct Mol Biol 31, 1251–1264 (2024). https://doi.org/10.1038/s41594-024-01274-x
[50] Antoine, L., Wolff, P. (2020). Mapping of Posttranscriptional tRNA Modifications by Two-Dimensional Gel Electrophoresis Mass Spectrometry. In: Arluison, V., Wien, F. (eds) RNA Spectroscopy. Methods in Molecular Biology, vol 2113. Humana, New York, NY. https://doi.org/10.1007/978-1-0716-0278-2_8
[51] McLuckey, S.A., Van Berkel, G.J. and Glish, G.L. (1992) Tandem mass spectrometry of small, multiply charged oligonucleotides. J Am Soc Mass Spectrom, 3, 60-70.
